# Two New Plasmid Post-segregational Killing Mechanisms for the Implementation of Synthetic Gene Networks in *Escherichia coli*

**DOI:** 10.1016/j.isci.2019.03.019

**Published:** 2019-03-22

**Authors:** Alex J.H. Fedorec, Tanel Ozdemir, Anjali Doshi, Yan-Kay Ho, Luca Rosa, Jack Rutter, Oscar Velazquez, Vitor B. Pinheiro, Tal Danino, Chris P. Barnes

**Affiliations:** 1 Department of Cell and Developmental Biology, University College London, London WC1E 6BT, UK; 2 Department of Genetics, Evolution and Environment, University College London, London WC1E 6BT, UK; 3 Centre for Mathematics, Physics and Engineering in the Life Sciences and Experimental Biology, University College London, London WC1E 6BT, UK; 4 Institute of Structural and Molecular Biology, University College London, London WC1E 6BT, UK; 5 Department of Biomedical Engineering, Columbia University, New York City, NY 10027, USA; 6 Data Science Institute, Columbia University, New York, NY 10027, USA; 7 Herbert Irving Comprehensive Cancer Center, Columbia University, New York, NY 10032, USA; 8 KU Leuven Rega Institute for Medical Research, Herestraat, 49 Box 1030, 3000 Leuven, Belgium

**Keywords:** Biological Sciences, Gene Network, Bioengineering

## Abstract

Plasmids are the workhorse of both industrial biotechnology and synthetic biology, but ensuring they remain in bacterial cells is a challenge. Antibiotic selection cannot be used to stabilize plasmids in most real-world applications, and inserting dynamical gene networks into the genome remains challenging. Plasmids have evolved several mechanisms for stability, one of which, post-segregational killing (PSK), ensures that plasmid-free cells do not survive. Here we demonstrate the plasmid-stabilizing capabilities of the axe/txe toxin-antitoxin system and the microcin-V bacteriocin system in the probiotic bacteria *Escherichia coli* Nissle 1917 and show that they can outperform the commonly used hok/sok. Using plasmid stability assays, automated flow cytometry analysis, mathematical models, and Bayesian statistics we quantified plasmid stability *in vitro*. Furthermore, we used an *in vivo* mouse cancer model to demonstrate plasmid stability in a real-world therapeutic setting. These new PSK systems, plus the developed Bayesian methodology, will have wide applicability in clinical and industrial biotechnology.

## Highlights

• axe/txe and microcin-V stabilize plasmids better than hok/sok, the current standard
• Microcin-V kills invading plasmid-free cells but is vulnerable to resistant mutants
• hok/sok and axe/txe improve plasmid stability in an *in vivo* tumor model

## Graphical Abstract

**Figure:**
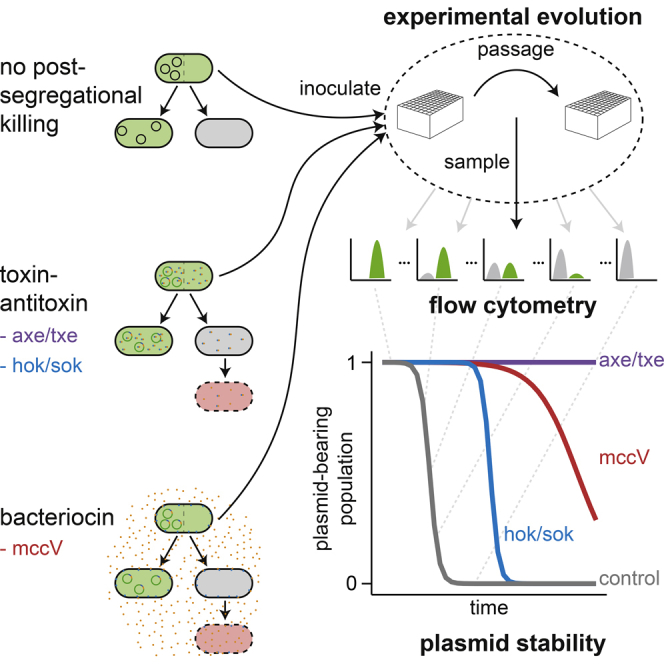

## Introduction

The genes comprising a synthetic circuit can be maintained in a host bacterium in two ways: on the chromosome of the organism or on extra-chromosomal material such as plasmids. Plasmids are a fundamental biological tool and have been widely used in molecular and cellular biology research, leading to a number of well-developed methods for their manipulation (Ellis et al., 2011, Casini et al., 2015). This ease of manipulation enables a level of modularity, which is one of the key engineering goals of synthetic biology (Andrianantoandro et al., 2006, Martinez-Garcia et al., 2014). Plasmid copy number can be an important parameter in the functioning of circuits, and it is non-trivial to convert a dynamical gene circuit from a multi-copy plasmid implementation to a single-copy implementation and maintain identical function (Lee et al., 2016).

**Figure 1:**
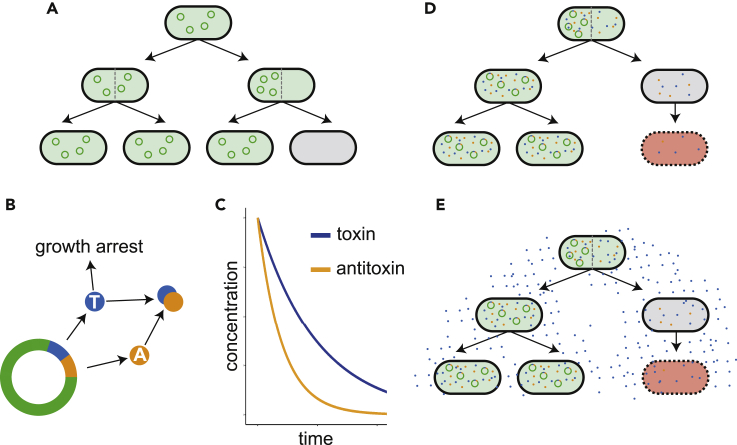
Plasmid Stability and Post-segregational Killing (A) Plasmid-free cells are produced due to uneven distribution of plasmids at cell division. (B) Toxin-antitoxin PSK systems are plasmid-borne mechanisms. (C) These systems rely on a long-lived toxin degrading more slowly in a newly plasmid-free cell than the antitoxin. (D) Post-segregational killing mechanisms carried on plasmids cause the death of cells that lose the plasmid when dividing. (E) Bacteriocins can improve plasmid stability by a population-level policing through the secretion of a toxin into the environment.

However, one of the fundamental problems with using plasmids is their segregational instability. When bacteria divide there is the possibility that all plasmid copies remain in one-half of the cell, which leads to the production of a plasmid-free daughter cell, as shown in Figure 1A. As most of the synthetic circuits that are borne on plasmids produce a burden to their hosts, the plasmid-free population outgrows the plasmid-bearing population and the engineered strain is quickly diluted from the environment (Summers, 1991). Maintaining the presence of the engineered circuit within the bacterial population is fundamentally important in the design of a predictable synthetic biological system.

Antibiotic selection of bacteria-containing plasmids with the corresponding antibiotic resistance genes is commonly used in a research environment. However, antibiotics are not used in industrial fermentation because of the financial impact of removal and deactivation (Kroll et al., 2010). Antibiotics are also unsuitable for clinical applications for a number of reasons including horizontal gene transfer of resistance genes (Stecher et al., 2012, Wright et al., 2008) and the disruption of the native microbiota (Theriot et al., 2014). In light of these limitations, efforts have already been made to reuse a variety of existing microbial mechanisms to ensure plasmid persistence in more complex environments (Kroll et al., 2010, Wright et al., 2013). Successful alternatives have been demonstrated with the use of toxin-antitoxin (TA) systems (Loh and Proft, 2013, Danino et al., 2015), active partitioning mechanisms (Danino et al., 2015, Liu et al., 2005), and auxotrophy (Velur Selvamani et al., 2014a, Velur Selvamani et al., 2014b).

TA systems are a type of post-segregational killing (PSK) mechanism that function through the production of a long-lasting toxin and its shorter-lived antitoxin (Cataudella et al., 2012, Diago-Navarro et al., 2013), both encoded on the plasmid (Figures 1B and 1C). While the plasmid is present in the cell, antitoxin is being produced to neutralize the toxin. If, however, a plasmid-free daughter cell arises, the antitoxin quickly degrades and is unable to be replaced due to lack of the necessary genes. The negative effects of the toxin are no longer prevented, leading to the killing or growth prevention of the cell (Figure 1D). The successful PSK of a plasmid-free cell is reliant on enough toxin becoming active in the cell before it goes on to divide again, further diluting the toxin. Just as with the distribution of plasmids giving a probability of producing plasmid-free cells, there is a probability that the distribution of toxin is such that the TA system fails to kill the newly plasmid-free cell. As such, TA systems are fallible, and once plasmid-free cells escape, they have no mechanism for preventing their growth and the consequent dilution of the plasmid-bearing population. hok/sok is a type I TA system originating from the *parB* locus of the *E*. *coli* plasmid R1 and has been shown to be effective in its native *E*. *coli* and the gram-negative *Pseudomonas putida* (Gerdes, 1988). axe/txe is a proteic, type II TA system originating from the axe*-*txe locus of the gram-positive *Enterococcus faecium* plasmid pRUM (Grady and Hayes, 2003). It has been demonstrated that axe/txe could be used to stabilize a luminescent reporter in the gram-positive *Enterococcus faecalis in vivo* without antibiotic selection for 5 days (Rosa et al., 2013). The axe/txe system was also found to be present in 75% of vancomycin-resistant enterococci isolates, a common hospital pathogen and growing global concern (Moritz and Hergenrother, 2007).

An alternative to TA systems are bacteriocins, which are bacterially secreted proteins that have a bactericidal effect on either a narrow or broad spectrum of other bacteria lacking immunity (Riley, 1998). By secreting these antimicrobial peptides, a plasmid-bearing population is able to police the environment, preventing the growth of plasmid-free cells (depicted in Figure 1E). Successful attempts have already been made to use bacteriocins, such as the Lcn972 system, to stabilize plasmids in *Lactococcus lactis* (Campelo et al., 2014) and the colicins A and E2 in *E*. *coli* (Inglis et al., 2013). Here we use the microcin-V system, which is encoded on conjugative plasmids in *E*. *coli*. It consists of a low-molecular-weight toxic bacteriocin, which kills through pore formation, along with the genes for an ABC transporter and immunity protein (Azpiroz and Laviña, 2007). Microcin-V does not undergo any post-translational modification and, as such, does not require any additional enzyme-encoding genes. The structure of the bacteriocin gene itself is also modular, which allows for its hybridization with other bacteriocins, enabling the targeting of other bacterial strains (Acuña et al., 2012, Acuña et al., 2015). Furthermore, it has been shown that *E*. *coli* Nissle (EcN) already carries two bacteriocin systems in its chromosome, microcins H47 and M (Grozdanov et al., 2004). More recently, it was also shown that native bacteriocins found in EcN were vital in mediating competition in the inflamed gut in an inter- and intraspecies manner (Sassone-Corsi et al., 2016).

In this work we develop a mathematical model that describes how PSK systems improve plasmid maintenance. Using several origins of replication, we show how plasmid copy number and burden affect plasmid stability, with and without a TA system. We use our mathematical model and develop a Bayesian inference procedure to quantify the efficacy of two PSK systems, axe/txe and microcin-V, in stabilizing a burdensome plasmid in EcN and compare them to the more commonly used hok/sok system. The model enables determination of PSK efficacy and also the extra metabolic burden due to the PSK system. We show that the bacteriocin, unlike the TA systems, is able to remove plasmid-free bacteria from an environment if they arise. We also investigate the ability of the PSK systems in stabilizing a luminescent reporter plasmid without antibiotic selection *in vitro* and in a mouse tumor xenograft model *in vivo*. Collectively, we show that the two new systems show a much greater potential for plasmid stabilization than hok/sok in EcN.

## Results

### A Mathematical Model for Plasmid Loss and Post-segregational Killing

Early mathematical models of plasmid stability consisted of terms for a plasmid-bearing population and plasmid-free population, growing exponentially at different rates with a constant probability of plasmid loss from the plasmid-bearing population (Boe et al., 1987). These models were used to determine plasmid loss rates from experimental data (Boe, 1996, Boe and Rasmussen, 1996) and extended to describe bacterial populations in a chemostat, introducing dilution as well as growth rates dependent on a substrate (Ganusov and Brilkov, 2002). A more detailed model of plasmid loss was devised that takes into account the age distribution of bacteria within a population, although the predictions produced were virtually indistinguishable from simpler models (Lau et al., 2013). However, this model did highlight the difficulties of trying to infer the plasmid loss rate from measured population data as, due to the exceedingly small plasmid loss rates of most systems, the growth rate differences dominate the dynamics and lead to overestimation of plasmid loss (Lau et al., 2013).

Here, we extend the early plasmid loss model (Boe, 1996) to include PSK by a TA system. In this model there are two populations, plasmid-bearing (*X*^+^) and plasmid-free (*X*^−^) populations

$$\frac{dX^{+}}{d\tau }=\gamma X^{+}-\lambda \gamma X^{+}$$

(Equation 1) $$\frac{dX^{-}}{d\tau }=X^{-}+\lambda \gamma X^{+}-\omega \lambda \gamma X^{+}$$

where *λ* is the probability of producing a plasmid-free daughter cell when a plasmid-bearing cell divides, *γ* is the ratio of plasmid-free doubling time to plasmid-bearing doubling time, and *ω* is the probability of successful PSK. The time step, *τ*, is equal to one plasmid-free generation.

Bacteriocins do not produce PSK in the traditional sense. Instead there is a constant killing pressure on the plasmid-free population rather than specific killing of newly plasmid free cells. As such, the equation for the change in plasmid-free population differs to take into account the different point at which the bacteriocin causes the death of plasmid-free cells.

(Equation 2) $$\frac{dX^{-}}{d\tau }=X^{-}+\lambda \gamma X^{+}-2\omega X^{-}$$

Figure S1 shows the effects of varying each of the model parameters on the dynamics of plasmid loss. The plasmid loss parameter, *λ*, which is related to average plasmid copy number, *n*, according to *λ* = 2^1−*n*^ (Summers, 1991), affects the gradient of the plasmid loss curves early on, when there are very few plasmid-free cells. As soon as a plasmid-free population is established, the doubling time ratio, *γ*, which is a measure of the burden placed on the cell by the plasmid, has a large effect on the gradient. For the TA model in Equation 1, the killing parameter, *ω*, has a similar effect on the parameter *λ*. This is because it acts to delay the establishment of a plasmid-free population. The killing parameter in the bacteriocin model produces different dynamics to that for the TA model as it acts on all plasmid-free cells rather than just newly plasmid-free cells. A hierarchical Bayesian method for fitting the model to data allows these parameters to be determined from simulated and experimental plasmid loss curves (Transparent Methods, Figure S2).

### Plasmid Copy Number and Burden Affect Plasmid Stability

**Figure 2:**
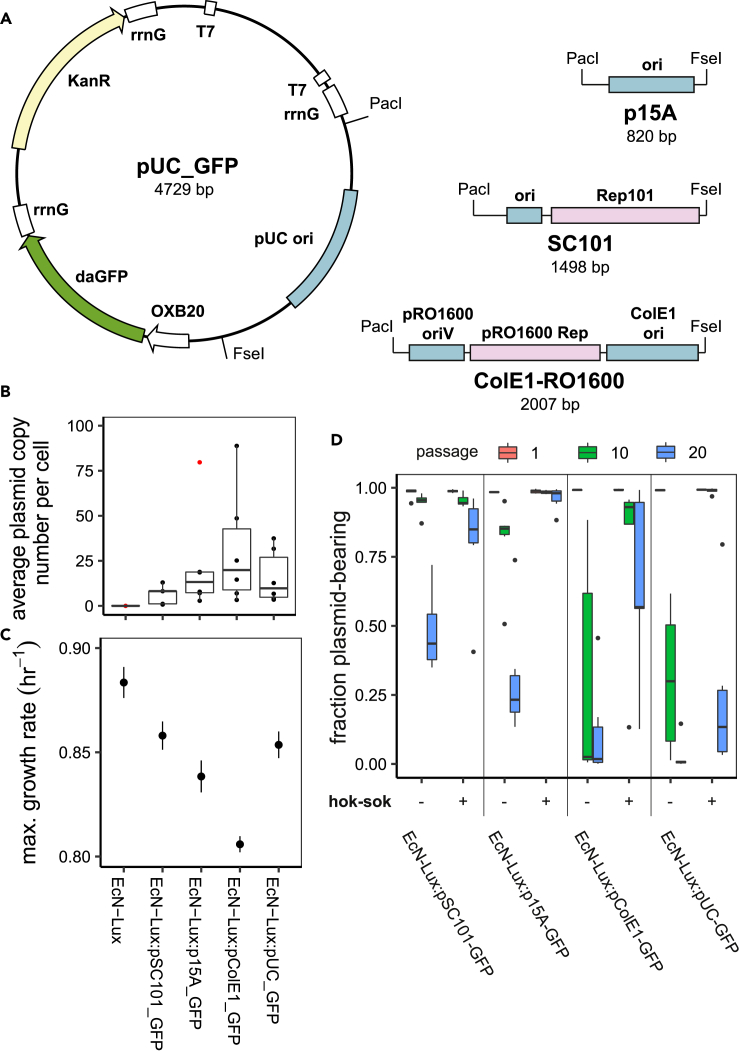
Plasmid Origin of Replication Affects Segregational Stability (A) Plasmids with different origins of replication were produced, constitutively expressing GFP. (B) Using qPCR, average plasmid copy number was calculated for each of the origins of replication. (Circles show samples, red circles are considered outliers.) (C) Changes in bacterial growth rate correspond to differences in plasmid copy number, with the higher-copy plasmids producing a larger reduction in maximal growth rate. (Filled circles show the mean calculated maximal growth rates, and lines show standard deviations.) (D) The effects of plasmid copy number and growth rate, dictated by the origin of replication, combine to alter the segregational stability of the different plasmids over 3 weeks of passaging. Although the commonly used hok/sok toxin-antitoxin system is able to increase stability, none of the plasmids are perfectly maintained over 20 days. (Black circles show outliers.)

A series of fluorescent reporter plasmids, expressing dasher GFP from the strong constitutive OXB20 promoter, were produced with four different origins of replication (Figure 2A), which would offer a range of plasmid copy numbers (Jahn et al., 2016). The SC101 origin, without native active partitioning system, provided a low plasmid copy number; p15A, a medium copy number; ColE1, a high copy number; and pUC, a very high copy number. Quantitative PCR (qPCR) was carried out to determine the copy number of these plasmids within EcN-Lux. Primers for plasmid amplification were chosen within the kanamycin resistance gene. This region is common across all the plasmids we used, negating the need to design primers for each plasmid. The qPCR results (Figure 2B) show that the SC101 origin does, indeed, produce the lowest plasmid copy number (∼4.8). The p15A origin has a slightly higher copy number (∼12), and the hybrid ColE1-RO1600 origin has an even higher copy number (∼21). However, the pUC origin is far from the very high copy number that has been reported in the literature previously (Yanisch-Perron et al., 1985) and is comparable with the p15A origin (∼14.5). Furthermore, the variance in copy number for the hybrid ColE1-RO1600 is large, with the highest copy replicate roughly four times higher than the median. These plasmid copy numbers are broadly in agreement with other recent estimates (Jahn et al., 2016).

Using a microplate reader to collect growth data and a non-parametric Gaussian process method to fit the growth curves (Swain et al., 2016), we were able to quantify the effect of carrying plasmids with different replication origins. The growth curves and model fits from which the maximal growth rates are estimated are shown in Figure S3. Growth rate measurements for each of the plasmids in EcN-Lux mirror the plasmid copy numbers measured using qPCR; the low copy SC101 plasmid has the lowest impact on growth rate, whereas the highest copy ColE1-RO1600 origin produces the greatest burden (Figure 2C). These results demonstrate the problem for plasmid stability; once the plasmid is dropped, the significantly higher growth in the plasmid-free strain causes the plasmid-bearing population to be outcompeted.

A daily passaging method was used to gather flow cytometry data, which was analyzed using an automated analysis pipeline (Transparent Methods, Figures S4–S11). This allowed us to determine plasmid stability over 21 days for the strains bearing plasmids with each of the four different replication origins, with and without the hok/sok TA system (Figure 2D). Our results are contrary to the conventional wisdom that a lower copy number plasmid will be lost sooner. Here we show that, in fact, the higher copy number plasmids, ColE1-RO1600 and pUC are lost sooner than the lowest copy SC101-based plasmid. This pattern is also reflected when the plasmids carry the hok/sok TA system. However, the p15A-based plasmid with the hok/sok system performs best, suggesting that there may be an optimum plasmid copy number for such TA systems.

### Toxin-Antitoxin Systems Minimally Impact Growth Rates

**Figure 3:**
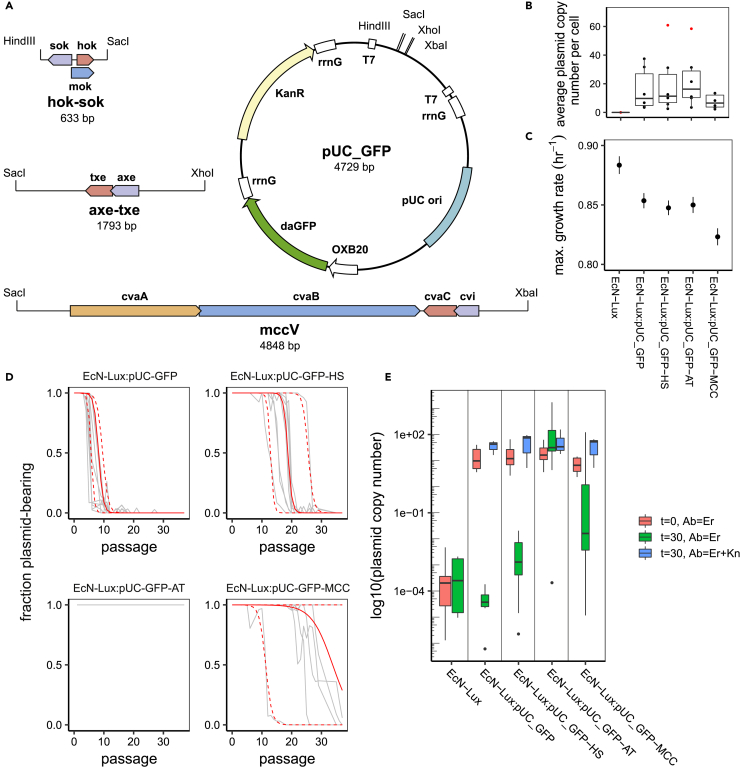
Plasmid Stability Mechanisms Have Varying Efficacy (A) Two toxin-antitoxin and one bacteriocin mechanism were cloned into the pUC-based burdensome plasmid and transformed into EcN-Lux. (B) qPCR shows that the two toxin-antitoxin systems have minimal effect on plasmid copy number. The bacteriocin system, however, seems to reduce copy number slightly. (Circles show samples, red circles are considered outliers.) (C) Maximal growth rates show that the burden of carrying the toxin-antitoxin systems does not affect growth. However, even with a lower copy number, the bacteriocin system shows a reduced maximal growth rate. (Filled circles show the mean calculated maximal growth rates, and lines show standard deviations.) (D) Plasmid loss curves for fluorescence-based plasmids in EcN-Lux. The gray lines show the trajectories of nine replicates for each strain. The solid red line shows the average model fit, and the dashed red lines show the 95% confidence intervals of the model with posteriors from all replicates. (E) qPCR carried out before undergoing passaging, after 30 days of passaging, and after 30 days of passaging with plasmid maintenance enforced by antibiotic selection. These back up the plasmid loss seen in the curves calculated from flow cytometry data (D). (Black circles show outliers.)

The pUC-based fluorescence reporter plasmid was used to determine the efficacy of three different plasmid-stability mechanisms: hok/sok, axe/txe, and microcin-V (Figure 3A). This plasmid was chosen as, from the above results, it resulted in the fastest plasmid loss of the four replication origins tested. qPCR was again used to determine the effect on plasmid copy number of carrying the different plasmid stability mechanisms (Figure 3B). This showed no difference between the control plasmid and the hok/sok-carrying plasmid. However, the axe/txe system shows a slight, but insignificant, increase in copy number, whereas the bacteriocin system shows a slight decrease in copy number.

Growth rates were assayed, and the maximal growth rates were determined (Figure 3C). The growth curves and model fits from which the maximum growth rates are estimated are shown in Figure S12. The maximal growth rates show a significant load on the growth rate between plasmid-free EcN-Lux and the plasmid-bearing strains. The TA systems have similar growth rates to the pUC-GFP control plasmid, repeating the pattern of plasmid copy number as an indicator of growth rate. However, the bacteriocin system, even with the slightly lower copy number measured from qPCR, has a slower growth rate than the other plasmids.

### Both axe/txe and Microcin-V Outperform hok/sok in Liquid Culture

Flow cytometry data (Figures S13–S16) were gathered using our daily passaging method and were used to derive plasmid loss curves for the pUC-GFP-based fluorescent plasmids in EcN-Lux over 37 daily passages (Figure 3D). For the control plasmid, without a PSK system (EcN-Lux:pUCGFP), the plasmid-free cells start to become apparent after only ∼4 passages, with the populations becoming entirely plasmid free after ∼12 passages. Figures 3D and S17 show that the TA systems improve the stability of the plasmid, although the commonly used hok/sok (EcN-Lux:pUC-GFP-HS) only provides stability for ∼16 passages, with one replicate beginning to drop as soon as passage 10 and one lasting until passage 22. Fitting our model to the data shows that the hok/sok system has a very high killing efficacy, with a survival probability of less than 5 × 10^−5^ (Figure S18) but still loses out to competition from the few plasmid-free cells that escape the toxin. Strikingly, axe/txe (EcN-Lux:pUC-GFP-AT) remains stable throughout the experiment, clearly demonstrating its efficacy over the other systems. As no plasmid loss is observed, the model-fitting procedure cannot be applied as there is no information on the probability of plasmid loss, *λ*. Finally, the results demonstrate that the microcin-V system (EcN-Lux:pUC-GFPMCC) outperforms the hok/sok system, although the results are varied, with four of the nine replicates remaining entirely plasmid bearing for the length of the experiment and in five of the replicates we see a plasmid-free population taking over. The model determined that efficiency of the bacteriocin killing is far below that of hok/sok, although, due to its population-scale effect, it still manages to produce stable populations for a longer period (Figure S18).

qPCR was carried out to determine how the plasmid copy number had changed, from the initial cultures to the cultures after 30 passages, in both the selective (erythromycin and kanamycin) and non-selective (only erythromycin) conditions (Figure 3E). After 30 passages of growth under selective conditions, the plasmid copy number had slightly increased for all the plasmids. This may be due to the enforced co-existence of the plasmids in the host leading to compensatory changes within the host or plasmid (Bouma and Lenski, 1988). The raw flow cytometry data show that when plasmid maintenance is enforced through the use of antibiotics the fluorescence levels remain stable across the full 37 passages for all but the microcin-V-bearing strain (Figures S13–S16). For the control plasmid and two TA system plasmids, this suggests that there is compensation between gene expression and copy number, which leads to the overall maintenance of fluorescence level. This conclusion is further supported by the population-level fluorescence data recorded using a microplate reader (Figure S17).

The average plasmid copy number after 30 passages of growth under non-selective conditions reinforces the results determined using flow cytometry. The control plasmid (EcN-Lux:pUC-GFP) has been completely lost, with levels comparable to the EcN-Lux strain with no plasmid. The hok/sok-carrying plasmid (EcN-Lux:pUC-GFP-HS) is similarly low, although there may be a small number of plasmids still present within the population that have not been removed through passaging. As was seen with the flow cytometry data, the axe/txe-bearing plasmid is still entirely present, and at levels comparable to the strains grown in selective media. Finally, the broad spread of copy numbers seen in the microcin-V system (EcN-Lux:pUC-GFP-MCC) reflects the heterogeneity seen in the flow cytometry data. Some of the replicates have completely lost the plasmid, whereas others are at an intermediate stage or are still plasmid bearing.

### Microcin-V Can Restore a Plasmid-Bearing Population

As we have seen with hok/sok, once plasmid-free cells arise in the population the TA system has no way to save the plasmid-bearing population. Bacteriocins, however, are able to police the entire population due to the toxin being secreted into the environment. This means that when plasmid-free cells arise or if the culture becomes contaminated, the bacteriocin system can push the population back toward entirely plasmid bearing.

**Figure 4:**
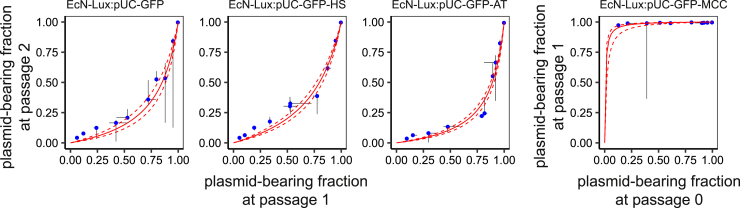
Plasmid-Bearing Strains Are Diluted with Plasmid-Free EcN-Lux at Different Ratios The dilutions are sampled after 24 h and show that the faster growth rate of the plasmid-free cells leads to further dilution of the plasmid-bearing population. In the case of EcN-Lux:pUC-GFP-MCC, however, the secreted bacteriocin allows the plasmid-bearing population to outcompete the plasmid-free cells. Blue points show the median of three replicates at each initial dilution; the bars show the minimum and maximum plasmid-bearing fraction at each passage. The solid red line shows the model fit, and the dashed red lines show the 95% confidence intervals. Each replicate was passaged once initially to allow for the cells to acclimatize to the growth conditions. However, for the EcN-Lux:pUC-GFP-MCC replicates, most plasmid-free cells were dead after one passage so the data from passage 0 to passage 1 were used.

Figure 4 shows that when a population of TA plasmid-bearing cells is diluted with plasmid-free EcN-Lux, the plasmid-free population outgrows the plasmid-bearing population, further diluting it. However, when we dilute the EcN-Lux:pUC-GFP-MCC cells, they quickly kill the plasmid-free population and restore the population. In fact, within 24 h there are no plasmid-free cells detectable in all but one anomalous sample.

### Microcin-V Instability Is Due to Immunity Development

As we have shown that the bacteriocin has the ability to kill plasmid-free cells introduced to their environment (Figure 4), why is there plasmid loss in Figure 3D? Three possibilities are the entire population is still plasmid bearing but some are no longer GFP expressing, the plasmid-bearing cells are no longer expressing the bacteriocin, or plasmid-free cells have developed immunity. To test these possibilities we used the cultures from the 30th passage of the plasmid loss experiment. These cultures were grown for 6 h with and without kanamycin selection. The cultures grown non-selectively reflect the state of the population at the time of being passaged. They were spotted next to a bacteriocin-producing, EcN-Lux:pUC-GFP-MCC, colony to determine if there was bacteriocin immunity in the population (Figure S19). All the colonies grew, indicating the presence of immune cells. Furthermore, fluorescence levels were not at the level of the colonies grown under kanamycin selection, indicating that it is not just plasmid-bearing cells surviving the bacteriocin. The cultures grown selectively were spotted next to bacteriocin-sensitive EcN-Lux colonies. The first point to note is that two of the colonies grew very little, suggesting almost complete loss of any residual plasmid-bearing cells from those cultures. Furthermore, the fluorescence levels of all the other colonies are greater than those of the colonies grown non-selectively. This indicates that plasmid-bearing cells, expressing GFP, are still present in these cultures but are diluted by kanamycin-sensitive, plasmid-free bacteria. Finally, for all but one of the colonies that grew under selective conditions, the growth of the bacteriocin-sensitive strain spotted next to them is inhibited. This shows that there are still bacteriocin-producing cells within those colonies. These observations together show that the plasmid loss seen in Figure 3D for the pUC-GFP-MCC-bearing strain is due to the development of immunity by plasmid-free bacteria (Feldgarden and Riley, 1999), not the loss of the ability to produce bacteriocin.

### axe/txe and hok/sok Successfully Stabilizes Luminescent Reporters *In Vivo*

**Figure 5:**
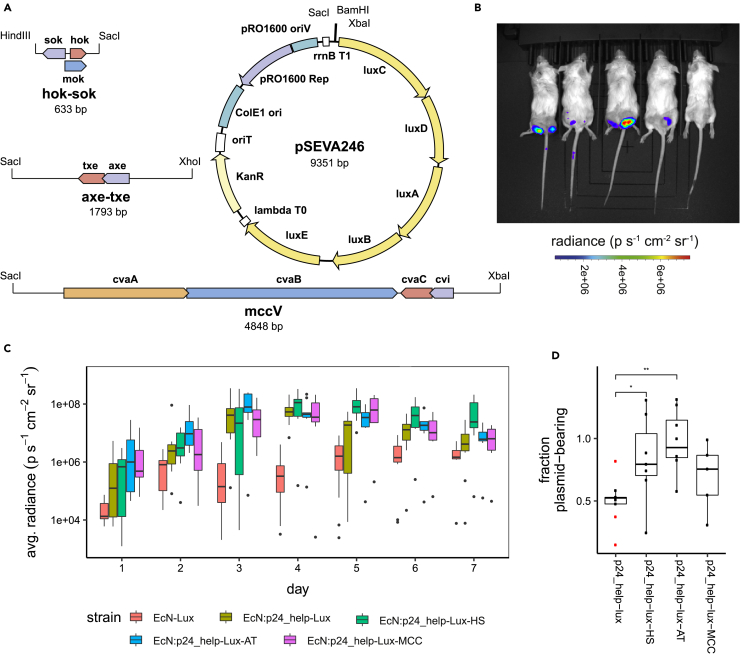
*In Vivo* Stability of Plasmid-Bearing EcN (A) The plasmid stability systems were cloned into a plasmid using the constitutive help promoter to express luminescence genes. (B) Representative image of plasmid-produced luminescence after bacterial colonization of implanted hind flank tumors. (C) The luminescence was measured for each tumor, once a day for a week, showing colonization of the tumors by the bacterial strain and continued production of the recombinant luminescence genes. (Black circles show outliers.) (D) Fraction of bacterial population remaining plasmid bearing 7 days after colonization, calculated from colony counts on selective and non-selective media performed in triplicate for each tumor. (Circles show samples, red circles are considered outliers. *p = 0.034, **p = 0.0013 in the Mann-Whitney U test for p24-Lux n = 9 tumors, p24-Lux-HS n = 7 tumors, p24-Lux-AT n = 8 tumors, p24-Lux-MCC n = 5 tumors.)

We produced a luminescent reporter plasmid, p24-Lux, that constitutively expresses the *luxCDABE* operon from the phelp promoter (Riedel et al., 2007) and cloned in the three PSK systems, so that we could visualize its presence *in vivo* (Figure 5A). Plasmid stability for these luminescent plasmids was then determined using the same protocol as for the fluorescent pUC-GFP-based strains. However, measurements could only be carried out using population luminescence in a microplate reader rather than single-cell fluorescence in a flow cytometer. Figure S20 shows that the control population (EcN:p24-Lux) has almost completely stopped luminescing by the end of the first passage. The two TA strains and the microcin-V-carrying strain perform better than the control and rank in the same order as the fluorescent plasmids. However, the luminescence loss occurs faster and even affects the axe/txe-bearing strain.

EcN strains containing the p24-Lux-HS, p24-Lux-AT, p24-Lux-MCC, or p24-Lux control constructs were intravenously injected into a mouse tumor model for *in vivo* characterization (Danino et al., 2015). Once administered, the EcN strain was shown to colonize the tumors in the mouse flanks within 3 days (Figure 5A). The mice were visualized daily, and luminescence readings were taken to determine the presence of protein production by our engineered bacteria within each tumor. Regions of interest (ROIs) were manually drawn around each tumor. The average radiance across each ROI (Figure 5C) shows the colonization and continued production of luminescence by the strains. The luminescence peaks between 3 and 4 days after administration and then begins to decline. However, the luminescence of tumors for mice administered with the EcN-Lux strain without plasmid, continues to increase, indicating that the continued presence of bacteria in the tumors is possible. This indicates that the decline in luminescence from the tumors in mice administered with the plasmid-bearing strains could be due to plasmid loss.

The tumors were then excised 7 days after injection, and a colony count was performed from the homogenized tissue to independently measure the stabilizing effect of the axe/txe, hok/sok, and microcin-V systems on the reporter construct. Figure 5D shows that without any stabilizing system ∼50% of the population had dropped the p24-Lux plasmid after intravenous injection and tumor colonization. In comparison, the p24-Lux-AT was significantly stabilized (p = 0.0013) with nearly 100% of the population maintaining the plasmids after colonization. The strain p24-Lux-HS also showed significant stability over p24-Lux control (p = 0.034) with ∼80% of the population maintaining the plasmids after colonization. The bacteriocin plasmid p24-Lux-MCC did not show a significant improvement over the control, although the mean plasmid-bearing population was ∼70%. There was also no significant difference between the plasmid-fraction-bearing population in p24-Lux-AT and p24-Lux-HS.

## Discussion

We developed an automated flow cytometry pipeline, mathematical model, and Bayesian inference procedure to quantify the efficacy of PSK systems stabilizing fluorescent and luminescent reporter constructs *in vitro* and *in vivo*. We used this pipeline to demonstrate that two new PSK systems in EcN, axe/txe and microcin-V, can outperform the more commonly used hok/sok system. Our method for Bayesian parameter estimation is able to accurately infer parameters from simulated data, and, where plasmid loss occurs, it can be used to estimate growth rate differences, plasmid loss rates, and PSK efficacy.

As expected, plasmids expressing fluorescent and luminescent proteins were shown to reduce the growth rate of EcN significantly, but there was minimal extra burden produced from the TA systems and a small burden from the bacteriocin system. Using four different plasmid origins of replication we have shown that plasmid copy number proportionally affects the growth rate of the plasmid-bearing strain. Here we have shown that the conventional wisdom, that lower copy number plasmids are less stable, is incorrect. Our mathematical model demonstrates that burden is an important consideration in plasmid stability. As such, any reduction in growth rate associated with higher copy number can prove more of a hindrance to plasmid maintenance than the increased chance of plasmid loss due to low plasmid copy number. Future work could include the use of whole-cell models to investigate how burden, copy number, and plasmid instability are related.

Our plasmid stability assays with both the fluorescent and luminescent plasmids showed that axe/txe provides greater plasmid stability than hok/sok. This is an interesting result as hok/sok is an *E*. *coli*-native TA system, whereas axe/txe was found in the gram-positive *E*. *faecium*. Furthermore, hok/sok is bactericidal (Faridani et al., 2006), whereas axe/txe is only bacteriostatic (Grady and Hayes, 2003). One might expect that the plasmid-free persister cells created from the txe toxin would begin to divide at a later passage and cause the plasmid-bearing population to become diluted, but this did not appear to happen during the 37 passages of the fluorescent strains.

The results for microcin-V show far more variability in plasmid stability than those for the TA systems. We have demonstrated that in those replicates in which plasmid loss was observed, plasmid-free cells had developed immunity to the bacteriocin. The similarity of microcin-V to bacteriocin systems already present in EcN 1917 may have made it simpler for such immunity to develop. However, there are a vast range of other bacteriocins to choose from that may well prove more reliable for prolonged applications.

Although microcin-V does not perform as well as axe/txe over the length of the plasmid stability experiments, we have shown that over a single passage of 24 h the bacteriocin is able to push a diluted population back to entirely plasmid bearing. For this reason, microcins may be particularly effective in industrial fermentation applications wherein the environment is relatively stable compared with the *in vivo* conditions encountered in clinical use.

A major focus of synthetic biology over the coming years will be to push proof-of-principle systems into real-world applications. A big limitation for industrial and clinical applications adopting synthetic biology tools is the use of antibiotic selection, and moving away from these laboratory-based systems will be critical. Our developed experimental and modeling approach can be used to characterize new systems for industrial applications and novel therapeutics. By inferring information about the relative growth rates and killing efficiency, a more quantitative viewpoint of plasmid stability mechanisms can be achieved, enabling the targeting of PSK systems to specific applications.

### Limitation of Study

Our study contains two main limitations, which could be addressed with further work. (1) The mathematical model assumes constant plasmid copy number, within the plasmid-bearing population, during a passage, and between all passages. The average plasmid copy number at different time points within a passage has been shown to be dependent on the plasmid origin of replication (Jahn et al., 2016). It is, however, commonly known that adaptation between plasmid and host bacteria occurs after several generations (Bouma and Lenski, 1988) and that regulation of plasmid copy number may be involved. (2) Our modeling also assumes no change in growth rate across passages, which due to the coevolution of plasmid and host, is likely to be an oversimplification as fitter cells are selected.

## Methods

All methods can be found in the accompanying Transparent Methods supplemental file.

## Data and Software Availability

The data reported in this paper is available in Mendeley data.

## Supplemental Information

Document S1. Transparent Methods, Figures S1–S25, and Tables S1–S4
