# Supplementary material for: Two New Plasmid Post-segregational Killing Mechanisms for the Implementation of Synthetic Gene Networks in Escherichia coli
Source: iScience. 2019 Mar 22;14:323–34. doi: 10.1016/j.isci.2019.03.019 (PMC6489366; doi:10.1016/j.isci.2019.03.019)
Supplement: Document S1. Transparent Methods, Figures S1–S25, and Tables S1–S4 [file mmc1.pdf]

ISCI, Volume *14*

Supplemental Information

Two New Plasmid Post-segregational Killing Mechanisms for the Implementation of Synthetic Gene Networks in *Escherichia coli*

Alex J.H. Fedorec, Tanel Ozdemir, Anjali Doshi, Yan-Kay Ho, Luca Rosa, Jack Rutter, Oscar Velazquez, Vitor B. Pinheiro, Tal Danino, and Chris P. Barnes

1 Transparent Methods

Analytical Solutions to Plasmid Stability Models

Analytical solutions to the mathematical models in the main text were calculated using Mathematica (Wolfram Research, Inc. n.d.). The proportion of the population that is plasmid-bearing after τ plasmid-free generations can be written for the TA model as:

$$\begin{aligned}\phi_\tau(\lambda,\gamma,\omega) &= \frac{X^+(t)}{X^+(t)+X^-(t)} \\ &= \frac{e^{(\gamma-\gamma\lambda)\tau}}{\nu e^\tau + e^{(\gamma-\gamma\lambda)\tau} + \frac{(e^\tau - e^{(\gamma-\gamma\lambda)\tau})\gamma\lambda(1-\omega)}{1-\gamma+\gamma\lambda}}\end{aligned} \tag{1}$$

and for the bacteriocin model as:

$$\phi_\tau(\lambda,\gamma,\omega) = \frac{e^{(\gamma-\gamma\lambda)\tau}}{\nu e^{(1-2\omega)\tau} + e^{(\gamma-\gamma\lambda)\tau} + \frac{(e^{(1-2\omega)\tau} - e^{(\gamma-\gamma\lambda)\tau})\gamma\lambda}{1-\gamma+\gamma\lambda-2\omega}} \tag{2}$$

where $\nu = \frac{X^-(0)}{X^+(0)}$. For the plasmid loss experiments we assume that the initial population is entirely plasmid bearing i.e. $\nu = 0$.

Modelling Noise in Plasmid Loss Curves

The experimental method used to determine plasmid stability relies on sampling a population of cells and classifying each one as plasmid-bearing or plasmid-free. Every one of the n cells sampled is equivalent to a Bernoulli trial where "success" is a cell being plasmid-bearing and the probability parameter corresponds to the proportion of plasmid-bearing cells in the culture from which the sample was drawn. As such, a hypergeometric distribution can be used to describe the number of plasmid-bearing cells in any sample. However, since the fraction of the total population being sampled is very small, $\sim 1/5000$, a binomial model is a reasonable approximation:

$$X^+ \sim \text{Binomial}(n,\phi) \tag{3}$$

where n is the number of cells sampled and ϕ is the true proportion of plasmid-bearing cells in the population from which the sample was drawn.

The binomial model can describe the noise introduced in sampling the plasmid loss experiments but does not incorporate any intrinsic biological noise, which will result in overdispersion of the data. The "true proportion of plasmid-bearing cells", ϕ, from which the sample was taken, is the parameter that is effected by this biological noise. The uncertainty in this parameter is traditionally modelled using a beta distribution and the binomial and beta model can be combined into a beta-binomial model:

$$X^+ \sim \text{Beta-Binomial}(n,\alpha,\beta) \tag{4}$$

where

$$\alpha = \phi\left(\frac{1}{\rho}-1\right) \tag{5}$$

$$\beta = \frac{\alpha - \alpha\,\phi}{\phi} \tag{6}$$

thus the parameter ρ is used to reflect the uncertainty in ϕ. Simulated plasmid loss curves can be produced by sampling r replicates from the beta-binomial model at t timepoints. The plasmid-bearing population fraction is then given by:

$$\hat{\phi}_{r,t}(\lambda,\gamma,\omega,\rho,n) = \frac{X^+_{r,t}}{n} \tag{7}$$

where $X^+_{r,t}$ are r replicate samples at t timepoints from the beta-binomial distribution in Equation 4. ϕ in Equations 5 and 6, is generated at each timepoint using either the TA model in Equation 1 or the bacteriocin model in Equation 2. The effects of the sample size, n, and the uncertainty parameter, ρ, are shown in Supplementary Figure S22.

Strains and Plasmid Construction

Lysogeny broth (LB) media and agar was used during propagation and cloning of bacteria. Assays were carried out in LB media and when antibiotic selection was applied, kanamycin was used at 25 µg/mL and erythromycin at 100 µg/mL. All DNA manipulations were performed in *E. coli* DH5α (NEB) or *E. coli* Mach 1. All final plasmid stability assays were performed either in *E. coli* Nissle 1917 (EcN) or in *E. coli* Nissle 1917 with a genomically integrated erythromycin-resistant and *luxCDABE* cassette (EcN-Lux) (Danino et al. 2015) (Table S1).

Table S1: related to Figures 2-5. *E. coli* strains and plasmids used in this study.

Strain or plasmid	Description	Source
E. coli		
DH5α	Used for cloning.	New England BioLabs
Mach 1α	Used for cloning.	Invitrogen
EcN	*E. coli* Nissle 1917.	Prof. Ian Henderson, UK
EcN-Lux	*E. coli* Nissle 1917 with chromosomal *luxCDABE* and erythromycin resistance.	(Danino et al. 2015)
Plasmids		
pKG1022	A plasmid carrying hok/sok from pPR633.	(Gerdes 1988)
pREG531	A plasmid carrying axe/txe from pRUM.	(Grady & Hayes 2003)
pHK11	A plasmid carrying microcin-V from pColV-K30.	(Gilson et al. 1987)
pSEVA471	SC101 ori; SpR.	(Martinez-Garcia et al. 2014)
pSEVA261	p15A ori; KanR.	(Martinez-Garcia et al. 2014)
pSEVA246	Hybrid pRO1600/ColE1 ori; KanR; promoterless *luxCDABE*.	(Martinez-Garcia et al. 2014)
pUC-GFP	High copy pUC ori; KanR; 4540bp; constitutive daGFP expression under the strong OXB20 promoter.	Oxford Genetics
pUC-GFP-HS	pUC-GFP with hok/sok cassette.	This study
pUC-GFP-AT	pUC-GFP with axe/txe cassette.	This study
pUC-GFP-MCC	pUC-GFP with microcin-V cassette.	This study
pSC101-GFP	pUC-GFP with the pUC ori replaced with SC101 from pSEVA471.	This study
p15A-GFP	pUC-GFP with the pUC ori replaced with p15A from pSEVA261.	This study
pColE1-GFP	pUC-GFP with the pUC ori replaced with hybrid pRO1600/ColE1 ori from pSEVA246.	This study
pSC101-GFP-HS	pSC101-GFP with with hok/sok cassette.	This study
p15A-GFP-HS	p15A-GFP with hok/sok cassette.	This study
pColE1-GFP-HS	pColE1-GFP with hok/sok cassette.	This study
p24-Lux	pSEVA246 with phelp promoter in front of the *luxCDABE* operon.	This study
p24-Lux-HS	p24-Lux with hok/sok cassette.	This study
p24-Lux-AT	p24-Lux with axe/txe cassette.	This study
p24-Lux-MCC	p24-Lux with microcin-V cassette.	This study

Fluorescent assay plasmids were constructed using the pUC-GFP plasmid containing a pUC high copy origin-of-replication, a kanamycin resistance cassette and a dasher GFP gene being constitutively expressed in high quantities by the OXB20 promoter (Oxford Genetics, UK). The SC101, p15A and ColE1-RO1600 origins-of-replication were PCR amplified from their respective SEVA plasmids using the primers P.SEVA.ORI.F and P.PacI.SEVA.ORI.R. The fragments were digested using PacI and and FseI along with the pUC-GFP plasmid. The digested vector gel extracted to remove the band for the pUC origin and ligated with the new origin-of-replication fragments to create pSC101-GFP, p15A-GFP and pColE1-GFP.

Plasmid pUC-GFP-HS was created by ligating the 633bp HindIII to SacI fragment digested from pKG1022 (Gerdes 1988) into the MCS of pUC-GFP digested with the same restriction enzymes. Hok/sok was cloned into the other copy number plasmids in the same way. Plasmid pUC-GFP-AT was created by ligating the 1390bp BamHI to SacI fragment digested from pREG531 (Grady & Hayes 2003) into the MCS of pUC-GFP digested with the same restriction enzymes. Plasmid pUC-GFP-MCC was created by PCR amplification of pHK11 (Gilson et al. 1987) with primers P.SacI.mccV.FOR and P.XbaI.mccV.REV to amplify the microcin-V fragment with >400bp upstream and downstream of the annotated genes. This fragment was then digested with XbaI and SacI and ligated into pUC-GFP which had been digested using the same restriction enzymes. These plasmids were transformed into EcN-Lux using a standard thermal-shock transformation method to produce EcN-Lux:pUC-GFP, EcN-Lux:pUC-GFP-HS, EcN-Lux:pUC-GFP-AT, EcN-Lux:pUC-GFP-MCC (Table S1).

Luminescent assay plasmids were constructed from pSEVA246, a plasmid based on the SEVA architecture (Silva-Rocha et al. 2012, Martinez-Garcia et al. 2014) with a hybrid pRO1600/ColE1 high copy origin-of-replication, a kanamycin resistance cassette, and a multiple cloning site (MCS) upstream of a promoterless *luxCDABE* operon. The plasmids were produced in two steps. First the PSK systems were cloned into the MCS of plasmid pSEVA246. The hok/sok fragment was PCR amplified from pUC-GFP-HS with primers P.HS.SacI.F and P.HS.BamHI.R. This fragment was then digested with SacI and BamHI and ligated into pSEVA246 digested

Table S2: related to Figures 2-5. Primers used for the construction of plasmids. Underlined sequences show restriction sites added to the amplified fragments. Upper case letters show the annealing sequence.

Primer	Sequence
oris	
P.SEVA.ORI.F	CGGTGCTCAACGGGAATC
P.PacI.SEVA.ORI.R	cacaccttaattAAATCCGCCGCCCTAGAC
PSKs	
P.SacI.mccV.FOR	tgacgcgagctcTGCCCTTCCCTAGAGAATCC
P.XbaI.mccV.REV	actgtctctagaGGGTCAGTGCAGAATTTTTA
P.HS.SacI.F	atctgagagctcTCCGGCCGAACAAACTCC
P.HS.BamHI.R	atgtcaggatccAAGGAGAAAGGGGCTACCG

with the same enzymes. pUC-GFP-AT was digested with SacI and BamHI and the 1.3Kbp axe/txe fragment was ligated into pSEVA246 digested with the same enzymes. pUC-GFP-MCC was digested with SacI, XbaI and ApaLI; the third enzyme was added to cut the unwanted fragment and enable the gel extraction of the 4.8Kbp microcin-V fragment. This was ligated into pSEVA246 which had been digested with SacI and XbaI. The constitutive phelp promoter (Riedel et al. 2007) was cloned upstream of the *luxCDABE* cassette using Gibson assembly (Gibson et al. 2009). EcN was transformed individually with these plasmids via heat-shock methods to produce EcN:p24-Lux, EcN:p24-Lux-HS, EcN:p24-Lux-AT, EcN:p24-Lux-MCC (Table S1).

Growth Rate Assays

For fluorescent assay plasmids, overnight cultures of each strain were grown in selective LB media for 16 hours and diluted 1/1000 into 10 mL of non-selective LB media. 200 µL from each culture was then transferred into 6 wells of a black clear-bottom 96-well microtitre plate (Greiner Bio-one, Germany) and sealed with a Breathe-Easy sealing membrane (Sigma-Aldrich, UK). The plate was then grown in a shaking TECAN Spark microplate reader at 37°C for 20 hours with OD_{600} readings taken every 10 minutes. Growth curves were fitted using a non-parametric Gaussian process method (Swain et al. 2016). The same protocol was used for luminescent assay plasmids, however, due to the instability of the control luminescent plasmid (p24-Lux), kanamycin was added to the media for all of the plasmid-bearing strains in order to ensure the growth rates of plasmid-bearing bacteria were being measured. The luminescent assay plasmids were all compared to liquid cultures of EcN grown in plain LB media with no added antibiotics. The growth data and fits are shown in Supplementary Figures S3, S12 and S24.

qPCR Experiments to Determine Plasmid Copy Number

Calibration curves

Cultures of EcN-Lux and the four pUC-GFP based strains were grown overnight in 5 mL LB media with the appropriate antibiotics in 15 mL Falcon tubes. Cells were harvested from the overnight liquid cultures by centrifuging at 4C for 10 min at 4000 rpm. Supernatant was removed and cell pellet resuspended in a modified QuantiLyse buffer (10 mM Tris-HCl pH8.2, 100 µg mL^{-1} Proteinase K, 5 µM SDS (Pierce et al. 2002), with the addition of 1 mg mL^{-1} Lysozyme and 0.5 µg mL^{-1} Polymyxin B; QLP), a protease-based lysis buffer to extract DNA (genomic and plasmid) from cells. Resuspended cells were then further diluted with QLP buffer in 1:5 serial dilutions from 1/1 (neat) to 1/3125 of original cell content, to be used to generate qPCR standard curves. Samples were incubated at 37°C for 30 min, 50°C for 30 min, denatured at 95°C for 10 min, and held at 18°C.

The NEB Luna Universal qPCR Master Mix kit (#M3003) was used as per protocol (20 µL reactions), with 2 µL of each serial dilution sample as template DNA. Primers YKH341 (5- CAGGAGGCTTTCGCATGATTGA-3) and YKH342 (5- TCAGAGCAGCCGATTGTCTG-3) were used to amplify a 112bp portion of the aph(3)II (KanR) gene on the plasmid DNA (P), whilst primers YKH343 (5-GGTCAAGTCACCACCACTGT-3) and YKH344 (5-GCTCTTGTTCCATCTGGCGA-3) amplified a 121bp fragment spanning the terB-tus genes on the genomic DNA (G). qPCR of the serial dilutions of plasmid and genomic DNA was carried out on the LightCycler 480 Instrument II System, set to detect SYBR Green I (excitation at bandpass 465 nm, half band width 24 nm; detection at bandpass 510 nm, half band width 20 nm). The programme conditions are

Table S3: related to Figures 2 and 3. qPCR thermal program

	Target (°C)	Hold (mm:ss)	Ramp rate (°C s^{-1})
Denaturation			
	95	02:00	4.40
Quantification			
	95	00:15	1.00
	60	01:00	1.00
Melt curve			
	60	00:15	4.40
	95	-	0.03
Hold			
	40	00:30	2.20

separated into four stages, for initial denaturation of DNA, cycles of amplification and product/SYBR Green quantification, melt curve analysis, and a final holding step (Table S3).

Crossing point (Cp) values (the cycle at which the amplified sample fluorescence first rises above background fluorescence; typically when 10^{11} to 10^{12} molecules are present in the reaction; Roche Diagnostics GmbH, 2008) generated in the quantification stage are plotted against the $\log_{10}$ of dilution ratio values to generate standard curves for plasmid and genomic DNA (Supplementary Figure S25). The standard curves are used to identify an appropriate dilution ratio for subsequent qPCRs in amplifying plasmid and genomic DNA and for calculating PCR efficiency. PCR efficiency, E, was calculated from the gradient, m, of each standard curve with the equation $E = 10^{(-1/m)}$. While a standard curve with $m = -3.32$ would be a perfect amplification reaction with $E = 2$, often the reactions have inefficiencies. Genomic DNA amplification efficiency was 1.84, and plasmid DNA amplification efficiency was 1.88.

The melt curve analyses generated by the LightCycler 480 Software was used to check for specificity in product amplification. P amplicons are expected to have a melting temperature (Tm) of 85°C, whilst G amplicons have Tm=80°C. qPCR products were further run on 3% agarose, 1mM lithium acetate gels to verify the results from the melt curve analyses.

Plasmid loss confirmation

The wells of two 96-well microtitre plates were filled with 200 µL of LB media with erythromycin. In the first plate, each of the wells was inoculated from glycerol stocks of the strains used to inoculate the initial plate for the plasmid stability assay, producing six replicates of each strain. In the second plate, each of the wells was inoculated from glycerol stocks made at the 30th passage of the plasmid stability assay. These plates were sealed with Breathe-Easy sealing membranes (Diversified Biotech) and incubated at 37°C for 16 hours with continuous shaking.

As before, cells were harvested by centrifuging at 4°C for 10 min at 4000 rpm, the supernatant was removed, and the pellets were resuspended in QLP buffer. Samples were diluted down to 1/25 of original cell content, and used as template with the NEB Luna Universal qPCR Master Mix kit (#M3003), as described previously. Again, all qPCR products were run on 3% agarose, 1mM lithium acetate gels to verify qPCR product specificity.

The average plasmid copy number was calculate using the expression

$$n = \frac{E_G^{Cp_G}}{E_P^{Cp_P}} \tag{8}$$

where E_G and E_P are amplification efficiencies of the genomic and plasmid PCRs and Cp_G and Cp_P are the crossing point values determined for each sample.

Plasmid copy number determination

Cultures of each of the strains containing the plasmids with different origins of replication were grown overnight in 5 mL LB media with erythromycin and kanamycin in 15 mL Falcon tubes. The wells of a 96-well microtitre plate were filled with 200 µL of LB media with erythromycin and kanamycin. Each of the wells was inoculated with 1 µL from the overnight cultures, for a total of six replicates per strain. The plate was sealed with a Breathe-Easy sealing membrane and incubated at 37°C for 16 hours with continuous shaking. The plate was then treated as above to determine average plasmid copy number for each well.

Plasmid Stability Assays in Liquid Culture

Assays were performed in LB media in sterile deep square 96-well (2.2mL) polypropylene plate. The edge wells were used for LB media controls so that the strains weren't affected by any potential plate edge conditions (Hall et al. 2013). The 96-well plate was split into two sections; one section with 500mL LB media containing erythromycin (100µg/mL) and kanamycin (25 µg/mL) and the second with LB media containing only erythromycin The erythromycin was used to prevent external contamination as the resistance gene is encoded on the chromosome of the EcN-Lux bacterial strain. The kanamycin is used to ensure plasmid maintenance, generating a plasmid-bearing control throughout the experiment.

For the plasmid stability assay with various PSK mechanisms based on the fluorescent reporter plasmid, three double selective wells for each strain (EcN-Lux:pUC-GFP, EcN-Lux:pUC-GFP-HS, EcN-Lux:pUC-GFP-AT, EcN-Lux:pUC-GFP-MCC) were inoculated by picking single colonies from selective LB agar plates. Six single selective wells were inoculated with EcN-Lux, as a plasmid-free control, by picking single colonies from selective LB agar plates. The plate was covered with a System Duetz breathable silicon sandwich (EnzyScreen, The Netherlands) and clamped down in a shaking incubator at 37°C and 350 rpm for 24 hours. This produced the time point 0 cultures. These cultures were then used to inoculate 1:500 a fresh plate, arranged with the same selective layout. Each double selective culture was used to inoculate 3 replicates for the assay of PSK systems in single selective media. This produced 9 replicates of each plasmid-bearing strain in single selective media and 3 in double selective media. The EcN-Lux plasmid-free control had 6 replicates in single selective media. Although the double selective media was inoculated with the EcN-Lux strain, as expected, no bacteria grew. This plate was covered and incubated as before. For subsequent passages the plate was replicated 1:500 into fresh media with a one-to-one mapping between wells.

At each passage, 200 µL of the overnight culture was transferred into a black clear-bottom 96-well microtitre plate (Greiner Bio-one, Germany) and absorbance (600 nm), fluorescence (ex=480 nm, em=540 nm), and luminescence were measured in a microplate reader. In addition, 1 µL of the overnight culture was transferred into a round-bottom 96-well microtitre plate containing 200 µL of PBS and the single cell fluorescence was measured using a flow cytometer.

For the plasmid stability assay with various PSK mechanisms based on the luminescent reporter plasmid, the assay was carried out as above but the initial inoculations were with the luminescent strains (EcN:p24-Lux, EcN:p24-Lux-HS, EcN:p24-Lux-AT, EcN:p24-Lux-MCC) and the plasmid free control was EcN rather than EcN-Lux. Furthermore, the assays could not be performed with erythromycin as the EcN bacterial host did not contain the resistance gene. In order to prevent external contamination in LB cultures without any antibiotic, the entire plate was additionally exposed to UV light for 40 minutes before inoculation at each passage (Sharma 2012). Further, only absorbance and luminescence could be measured so the preparation of the plate for flow cytometry was not carried out. Absorbance was additionally measured at 410nm.

For the plasmid stability assay with various origins-of-replication, the assay was performed as with the fluorescent PSK plasmids. The initial inoculations were with strains EcN-Lux:pUC-GFP, EcN-Lux:pUC-GFP-HS, EcN-Lux:pSC101-GFP, EcN-Lux:pSC101-GFP-HS, EcN-Lux:p15A-GFP, EcN-Lux:p15A-GFP-HS, EcN-Lux:pColE1-GFP, EcN-Lux:pColE1-GFP-HS into double selective media and the plasmid free control was EcN-Lux. Due to the larger number of strains, each double selective culture was used to inoculate 2 replicates rather than 3.

Flow Cytometry Data Processing

In order to ensure the capture of all bacterial events during flow cytometry sampling, a threshold was used that also captured a considerable amount of non-bacterial events. This can be problematic as the small size of bacteria leads to the bacterial population appearing very close to this debris. A further problem is that more than one bacterium can be read as a single event due to clumping. A computational pipeline was developed to automate the gating of singlet bacteria while excluding background debris and bacterial doublets. This process is important to automate as it removes operator bias associated with the manual drawing of gates and speeds up the analysis when large numbers of samples are recorded.

The method fits two dimensional mixture models of one and two clusters to the forward-scatter-height and side-scatter-height variables with 95% confidence. Using integrated completed likelihood (ICL) (Biernacki et al. 2000) the number of clusters that best fits the data is determined. This allows for compatibility with experiments in which debris has been removed by hand by an experimenter or if not many debris events are recorded. For any clusters found, their median positions are determined and if a cluster is below a pre-determined threshold it is considered debris and removed.

Doublets are then removed in one of two ways. Initially, a linear model was fitted to the side-scatter-height

versus side-scatter-area data and points falling too far away were removed as doublets. An update to the Attune NxT software implemented area scaling which is calibrated automatically during the start-up procedure. The area scaling allows the assumption that singlets will lie on, or close to, the line side-scatter-height = side-scatter-area. As such, for later experiments this method is used for doublet discrimination.

The output from this processing are standard flow cytometry .fcs files with debris and doublets removed. The software, written in R, for performing this analysis can be found at https://github.com/ucl-cssb/autoGate.

Hierarchical Bayesian Model Fitting to Plasmid Loss Curves

The models detailed by Equations 1 and 2 are fitted to the experimental plasmid loss curves using a hierarchical Bayesian model.

From the plasmid loss experiments, at each timepoint, t, there are r replicates for which analysis of the flow cytometry data produces the number of plasmid-bearing cells $X^+_{r,t}$ in a sample of size $n_{r,t}$. Note that the sample size varies for each sample as all cells within a specified volume are measured rather than cutting off after a given number of events have been recorded.

The aim here is to determine the set of model parameters, $\{\lambda, \gamma, \omega\}$, and noise parameter, ρ, which are most likely to produce the data observed using flow cytometry. To do so we build a hierarchical model for the three plasmid loss model parameters, Supplementary Figure S23. In this model, the log of the plasmid loss probability, λ, has a global distribution for all strains with plasmids based on the same backbone. The posterior distribution for this global λ is determined by sampling from a uniform prior, $U(-10, 0)$ and the posterior distribution for the standard deviation, σ^λ, in the global λ is sampled from an exponential prior, $Exp(100)$, which produces a shrinkage pressure on the distribution. Below the global level, there is a distribution on λ for each strain, sampled from a normal distribution with a mean sampled from the global distribution on λ and a standard deviation sampled from the global distribution on σ^λ. There is a strain level standard deviation on the strain level λ which is sampled from an exponential prior in the same manner as the global one. Finally, at the lowest level, each replicate of a strain has a posterior distribution on λ which is sampled from a normal distribution with mean sampled from the strain level λ and standard deviation sampled from the strain level standard deviation.

Since the growth rates of each strain can be directly measured, strain level distributions for the growth ratio parameter, γ, can be directly determined. As such, the replicate level posterior distributions for γ are sampled from a normal distribution with mean sampled from the strain level γ and standard deviation given by the standard deviation of the strain level γ.

The parameter encapsulating the efficacy of the killing mechanisms, ω, cannot be said to be globally linked. This is particularly true when considering the differences between the bacteriocin and TA systems but also applies between different TA systems as they function in distinct ways. As such, each strain has a posterior distribution on ω and standard deviation sampled from uniform priors. Each replicate then has a posterior distribution on ω sampled from a normal distribution with mean and standard deviation sampled from the the strain level ω and standard deviation.

The fitting is performed for all data for all strains concurrently using Rstan, the R language version of stan (Carpenter et al. 2016). As such, the log likelihood can be written as:

$$L(\theta|\mathbf{X}^+) = \sum^{s}\sum^{r}\sum^{t} \log\left(P\left(X^+_{s,r,t}|\theta\right)\right) \tag{9}$$

where θ is the full set of parameters, $\{\lambda_{s,r}, \lambda_s, \sigma^\lambda_s, \lambda, \sigma^\lambda, \gamma_{s,r}, \gamma_s, \sigma^\gamma_s, \omega_{s,r}, \omega_s, \sigma^\omega_s, \rho_{s,r}\}$ and:

$$\begin{aligned} P(X^+_{s,r,t}|\theta) &= \text{Beta-Binomial}\left(n_{s,r,t}, \alpha_{s,r,t}, \beta_{s,r,t}\right) \\ \alpha_{s,r,t} &= \phi_{s,r,t}\left(\lambda_{s,r}, \gamma_{s,r}, \omega_{s,r}\right)\left(\frac{1}{\rho_{s,r}} - 1\right) \\ \beta_{s,r,t} &= \frac{\alpha_{s,r,t} - \alpha_{s,r,t}\ \phi_{s,r,t}\left(\lambda_{s,r}, \gamma_{s,r}, \omega_{s,r}\right)}{\phi_{s,r,t}\left(\lambda_{s,r}, \gamma_{s,r}, \omega_{s,r}\right)} \end{aligned}$$

This hierarchical method of fitting can be demonstrated using simulated data of two strains, with and without PSK. Both of these data sets are simulated using the same plasmid loss probability, λ, growth rate ratio, γ, and noise, ρ. The difference is in the PSK efficacy parameter, ω. Supplementary Figure S2 shows the model fit to the full set of timecourses for each simulated strain, using the strain level parameter posteriors.

Plasmid-bearing Strain Dilution Assays

Starter cultures were produced in the same way as for the plasmid stability assays. Different dilutions of plasmid-bearing to plasmid-free strains were then produced by pipetting different volumes of plasmid-bearing overnight culture into EcN-Lux overnight culture in percentages from 10% through to 100%, keeping a total volume of 100 µL. For example, to produce a 30% culture 30 µL of plasmid-bearing overnight culture was added to 70 µL of EcN-Lux overnight culture. Note that the overnight cultures were not normalised to the same optical density, so a 30% culture does not mean that 30% of the cells are plasmid-bearing. Assays were performed in LB media in sterile autoclaved deep square 96-well (2.2mL) polypropylene plate. Each well was filled with 500 µL of LB media with erythromycin (100 µg/mL). The wells were then inoculated in a random pattern with 3 replicates of each of the diluted cultures. At the same time a 96-well mictrotitre plate was prepared with 200 µL of PBS in each well and inoculated from the same diluted cultures and measured using the same flow cytometry set up as above. The deep well plate was then sealed and incubated as above. After 24 hours the plate was taken from the incubator and passaged while a flow cytometry plate was also prepared and measured. This was carried out for a total of 2 passages. We used the data from passage 1-2 for the model fitting as this allowed the cells time to get used to growing in the deep-well plate. However, for the MCC strain we had to use data for passage 0-1 because after 24 hours all plasmid-free bacteria had been killed.

Luminescent Plasmid Stability Assays *in vivo*

All animal work was approved by the institutional committee on animal care (Columbia, AC-AAAN8002). The protocol requires animals to be euthanized when tumours reach 2 cm^3, or under veterinary staff recommendation. The cell line (MC26-LucF, Tanabe laboratory, Massachusetts General Hospital) was obtained from, and authenticated by, the Tanabe laboratory, MGH. The cell line was tested to be mycoplasma-free before implantation in mice. Sample sizes for mice were determined by expected effect size to produce a power of 0.8-0.9. Mice were blindly randomized into various groups using a random number generator.

Animal experiments were performed on 6-week-old female BALB/c mice (Taconic Biosciences) with bilateral subcutaneous hind flank tumours from an implanted mouse colon cancer cell line (MC26-LucF). The concentration for implantation of the tumour cells was 10^8 cells per ml in DMEM (no phenol red). Cells were then implanted subcutaneously at a volume of 100 µL per flank, with each implant consisting of 10^7 cells. The MC26 cells where given 14 days to graft and grow until they reached a size of approximately 150-200 mm^3. Along with an EcN-Lux strain as a control, EcN:p24-Lux, EcN:p24-Lux-HS, EcN:p24-Lux-AT and EcN:p24-Lux-MCC were grown up in LB with kanamycin until exponential phase, washed three times in sterile PBS and then injected intravenously at a dosage of $1x10^6$ bacteria in 100 µL. After bacterial colonisation of the tumours, animals were monitored via IVIS (IVIS 200, Caliper Life Sciences) to detect the presence of the plasmid via bioluminescence. Briefly, this involved anaesthetising the mice with isoflurane and imaging with an open filter on the auto-exposure setting. After 7 days, each of the tumours were sterilely extracted and homogenised using a tissue dissociator (Miltenyi), an aliquot of which was seeded on each of LB plates and LB with kanamycin plates. The ratio was calculated by comparing the mean counts of plasmid bearing colonies on LB plates and LB plates with kanamycin from each tumour (Danino et al. 2015). ggplot2 package in R was used for plotting. Statistical significance was demonstrated by the Mann-Whitney U test with R.

Bacteriocin Resistance Check

The wells of a 96-well microtitre plate was filled with 200 µL of LB media, half with erythromycin and half with erythromycin and kanamycin. The wells of this plate were inoculated with 1 µL of each of the EcN-Lux:pUC-GFP-MCC wells from a glycerol stock of the 30th passage of the plasmid loss experiment, replicated so that there was a sample of each well growing under each selective condition. Additionally, wells of EcN-Lux and a control EcN-Lux:pUC-GFP-MCC were inoculated under each selective condition. This plate was then sealed with a Breathe-Easy membrane and grown at 37 °C in a shaking plate reader for 6 hours. 1 µL from each of the wells was then spotted onto an LB agar plate, also containing erythromycin. Next to each of the cultures grown in LB+Er was spotted 1 µL of the control EcN-Lux:pUC-GFP-MCC, grown in LB+Er. Next to each of the cultures grown in LB+Er+Kn was spotted 1 µL of EcN-Lux grown in LB+Er. The left to dry until the spots were no longer visibly wet. The plate was then placed in an incubator at 37 °C fro 16 hours. After incubation, images of the plate were taken on a blue light transilluminator with amber filter (IORodeo), to record GFP fluorescence, and using a gel documentation system with UV transillumination, to visualise colony growth (Supplementary Figure S19).

2 Supplementary Figures

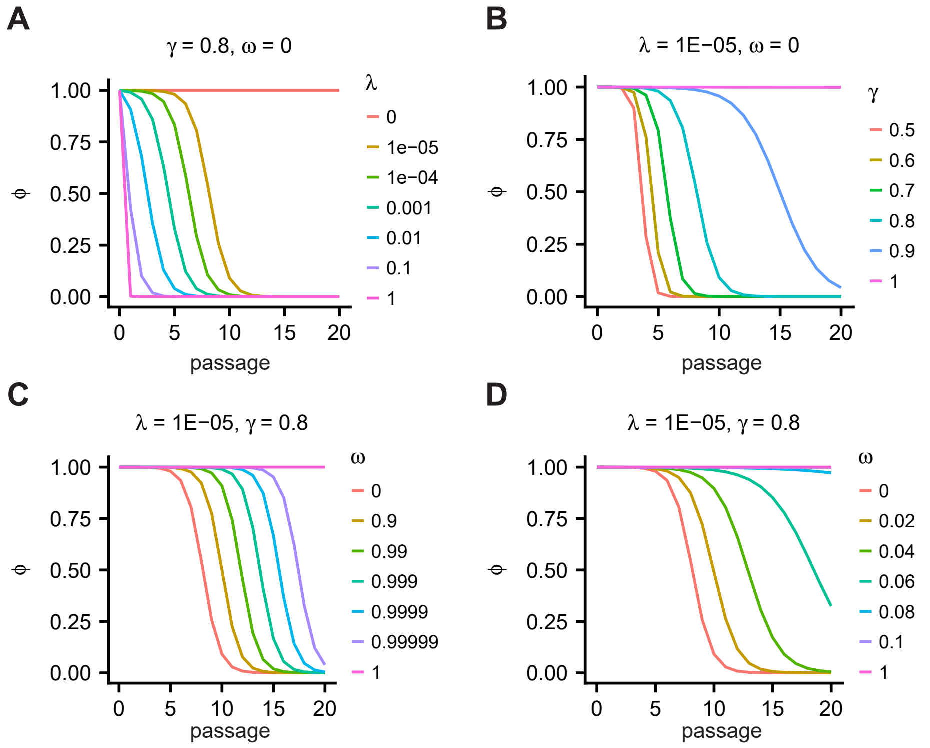

Figure S1: related to Figures 3 and 4. Effects of model parameters on plasmid-loss curves. A) Varying the plasmid loss probability, λ, and B) the doubling time ratio, γ, without post-segregational killing or bacteriocin killing. These are identical for the TA and the bacteriocin models when $\omega = 0$. C) Varying the efficacy of the toxin, ω, in the TA model. D) Varying the efficacy of the bacteriocin, ω, in the bacteriocin model.

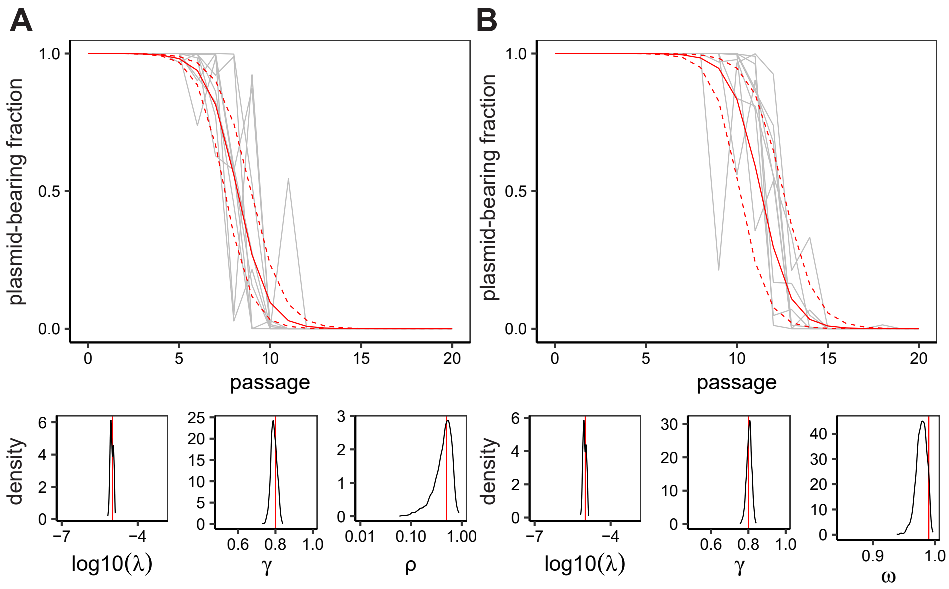

Figure S2: related to Figure 3. Fits of the hierarchical model at the strain level to simulated plasmid loss curves A) without PSK and B) with PSK. The grey lines show the nine simulated trajectories, the solid red line shows the mean model fit and the dashed lines show the 95% confidence intervals. Since the ω parameter is not relevant to the fitting of the timecourses without PSK, the posterior distribution of the noise parameter ρ is shown below the plasmid loss curve of (A) instead.

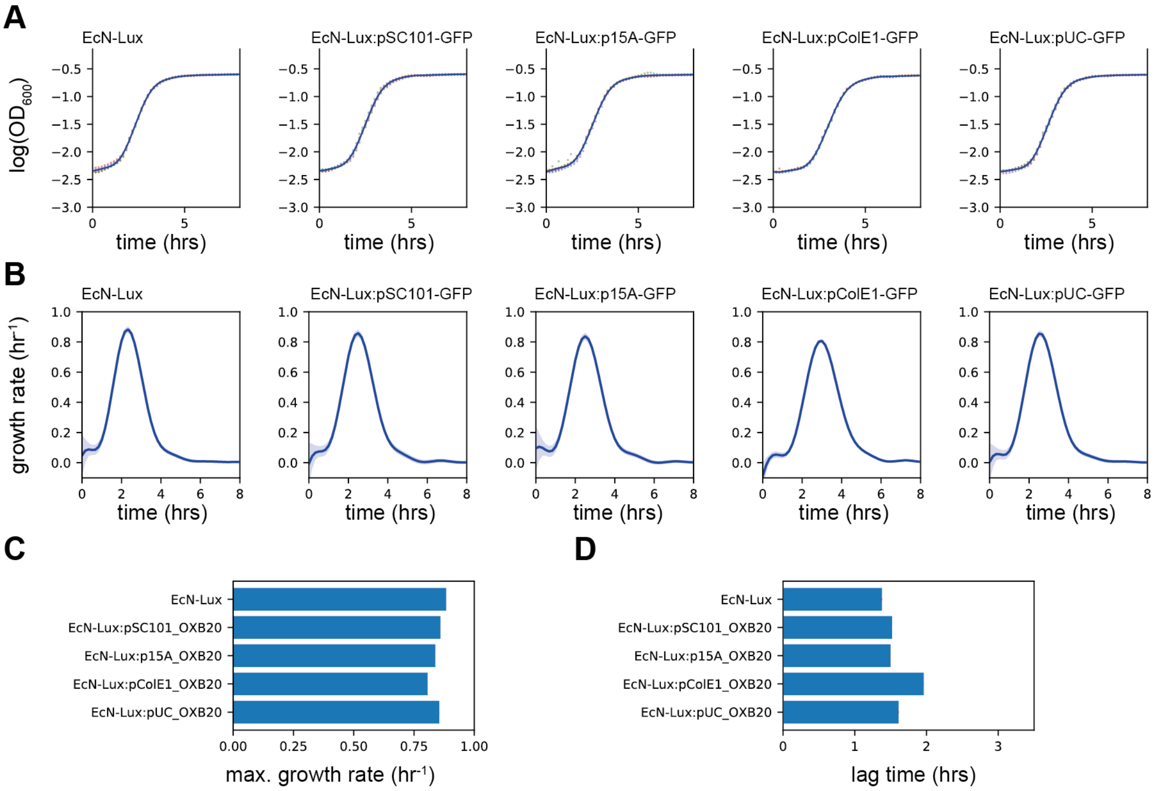

Figure S3: related to Figure 2. Growth curves and model fits for the fluorescent plasmids with different origins-of-replication in EcN-Lux grown in LB media with erythromycin. (A) Logged optical density measured at 600nm. The points show 6 replicates for each strain with the different colours indicating each replicate. The blue line shows the non-parametric Gaussian process fit with standard error shown by the light blue area around the line. (B) Estimated growth rate as a function of time. (C) Estimated maximal growth rates and (D) estimated lag time. The red lines show the standard deviation of the mean.

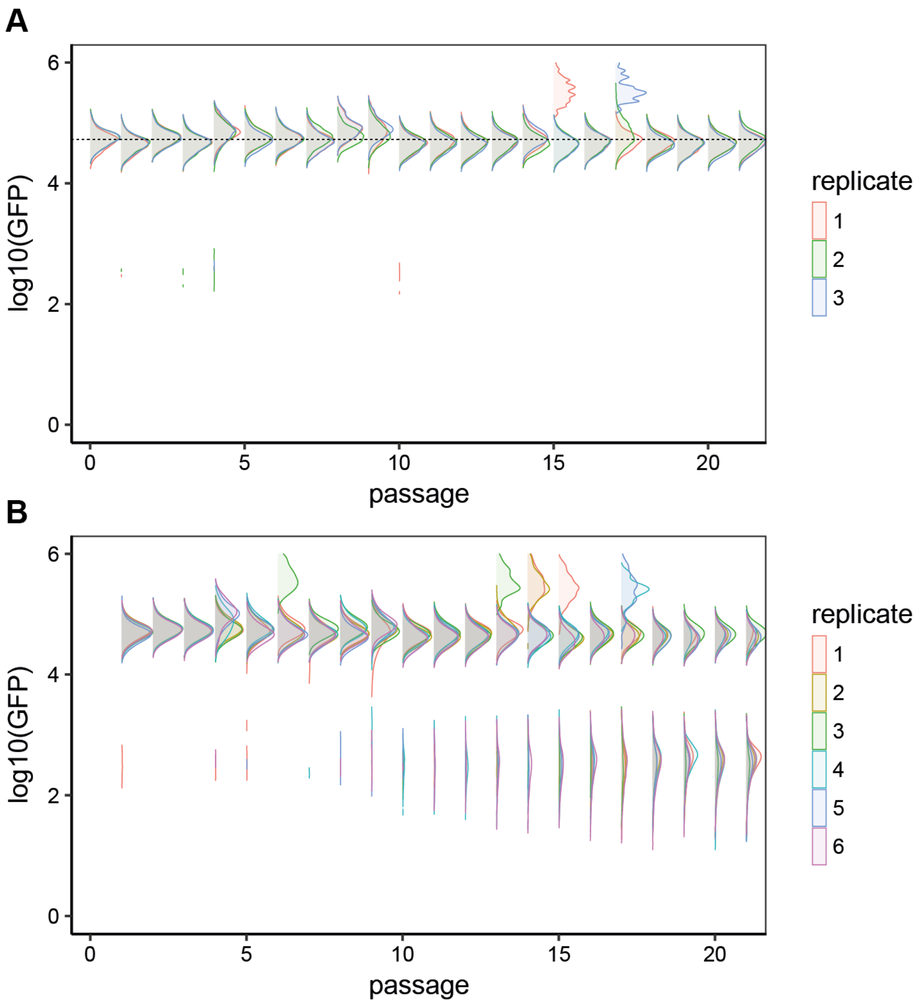

Figure S4: related to Figure 2. Flow cytometry samples of EcN-Lux:pSC101-GFP. A) 3 replicates grown in LB media with erythromycin and kanamycin, ensuring plasmid maintenance. The black dashed line indicates the median fluorescence at passage 0. B) 6 replicates grown in LB media with erythromycin only, allowing for plasmid loss.

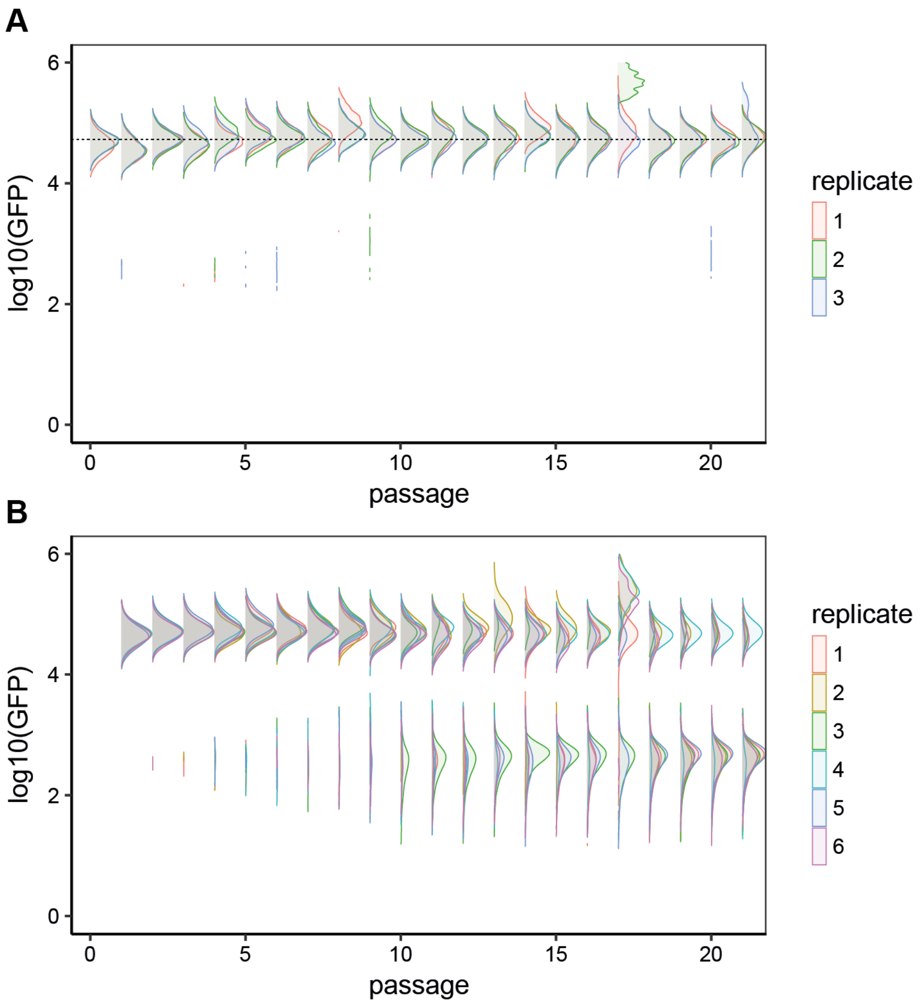

Figure S5: related to Figure 2. Flow cytometry samples of EcN-Lux:p15A-GFP. A) 3 replicates grown in LB media with erythromycin and kanamycin, ensuring plasmid maintenance. The black dashed line indicates the median fluorescence at passage 0. B) 6 replicates grown in LB media with erythromycin only, allowing for plasmid loss.

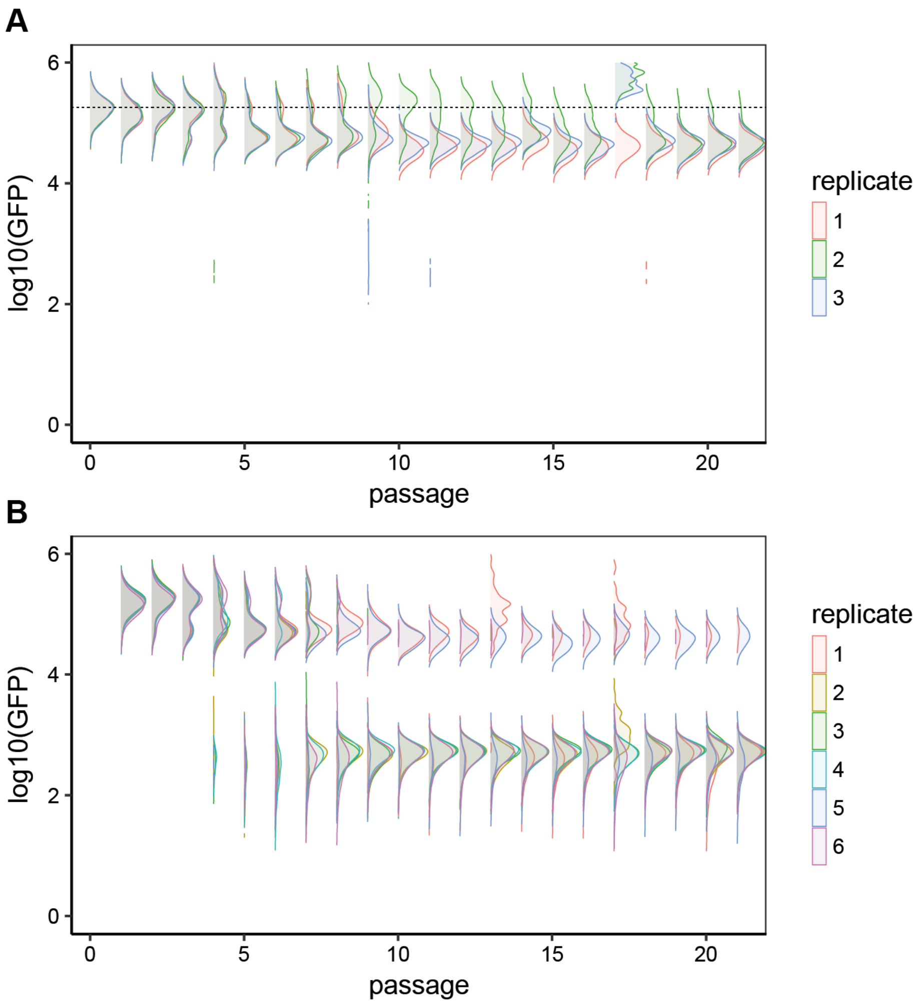

Figure S6: related to Figure 2. Flow cytometry samples of EcN-Lux:ColE1-GFP. A) 3 replicates grown in LB media with erythromycin and kanamycin, ensuring plasmid maintenance. The black dashed line indicates the median fluorescence at passage 0. B) 6 replicates grown in LB media with erythromycin only, allowing for plasmid loss.

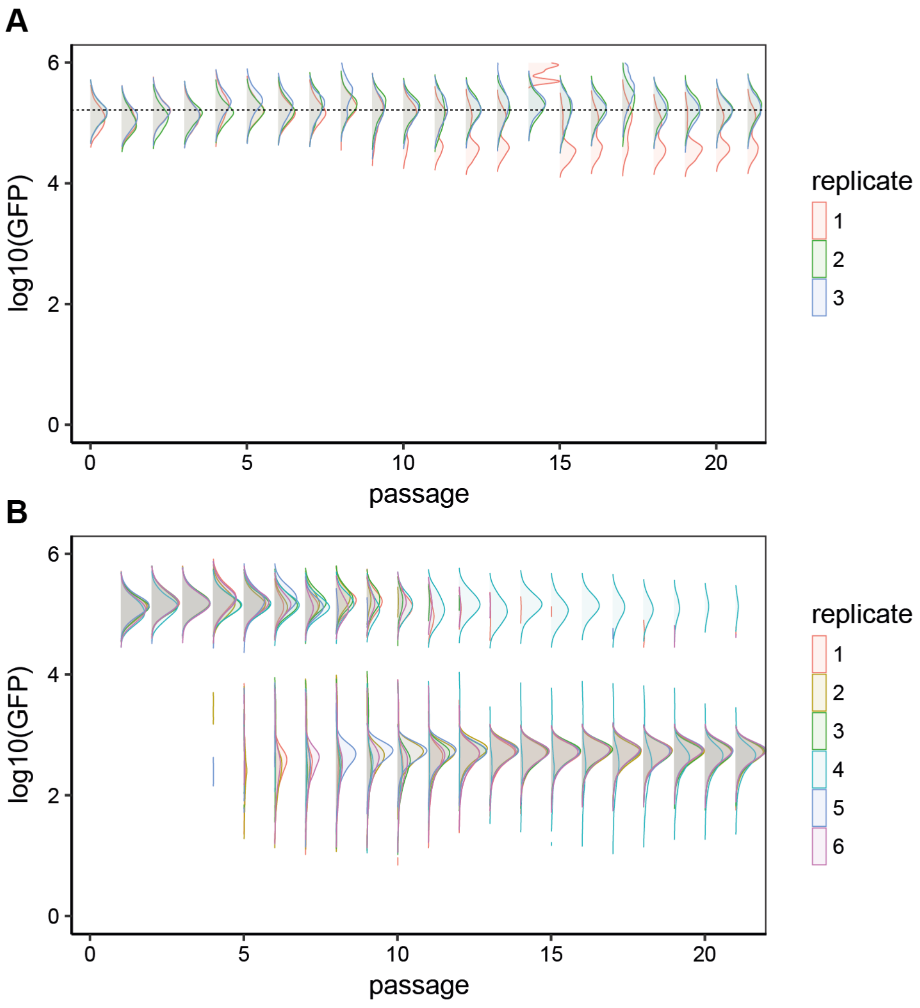

Figure S7: related to Figure 2. Flow cytometry samples of EcN-Lux:pUC-GFP. A) 3 replicates grown in LB media with erythromycin and kanamycin, ensuring plasmid maintenance. The black dashed line indicates the median fluorescence at passage 0. B) 6 replicates grown in LB media with erythromycin only, allowing for plasmid loss.

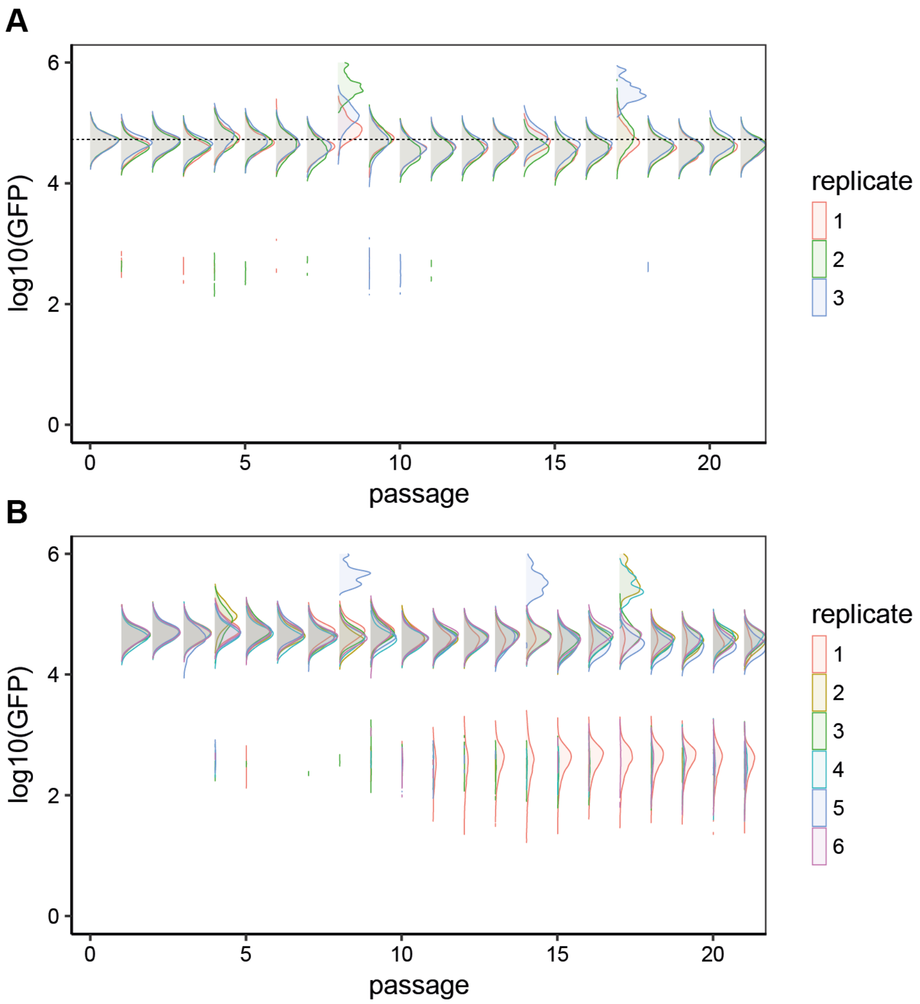

Figure S8: related to Figure 2. Flow cytometry samples of EcN-Lux:pSC101-GFP-HS. A) 3 replicates grown in LB media with erythromycin and kanamycin, ensuring plasmid maintenance. The black dashed line indicates the median fluorescence at passage 0. B) 6 replicates grown in LB media with erythromycin only, allowing for plasmid loss.

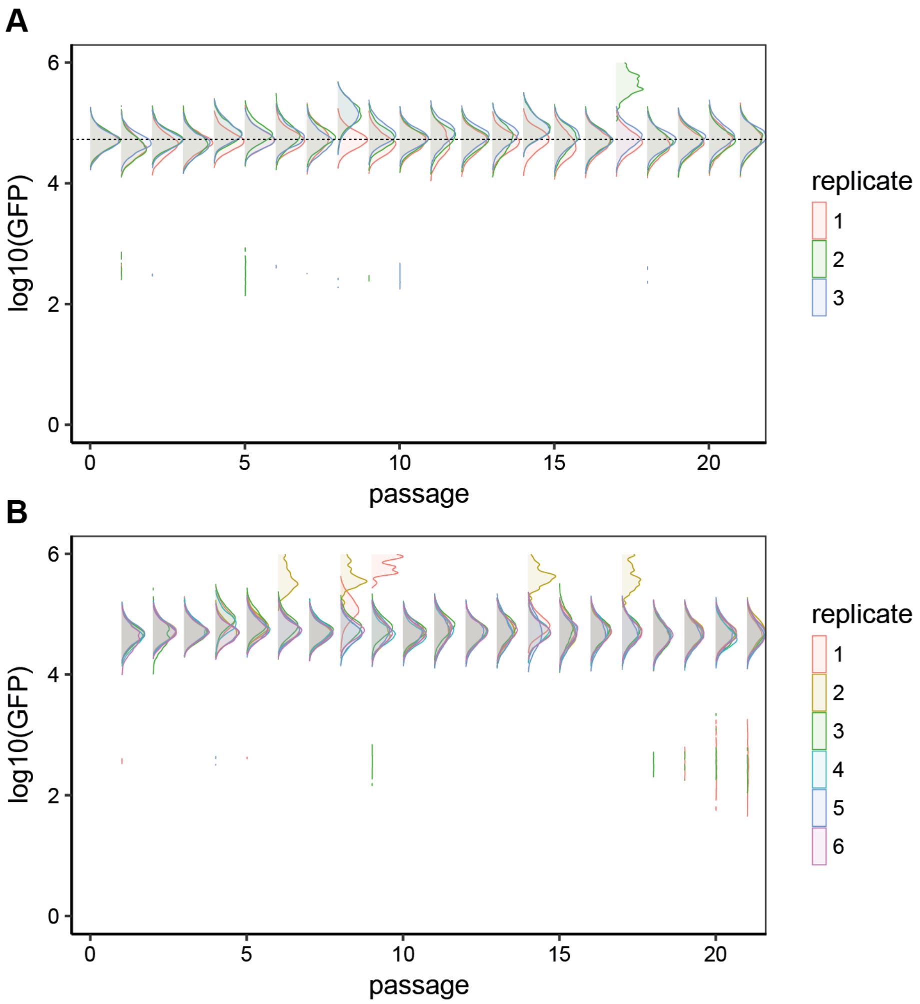

Figure S9: related to Figure 2. Flow cytometry samples of EcN-Lux:p15A-GFP-HS. A) 3 replicates grown in LB media with erythromycin and kanamycin, ensuring plasmid maintenance. The black dashed line indicates the median fluorescence at passage 0. B) 6 replicates grown in LB media with erythromycin only, allowing for plasmid loss.

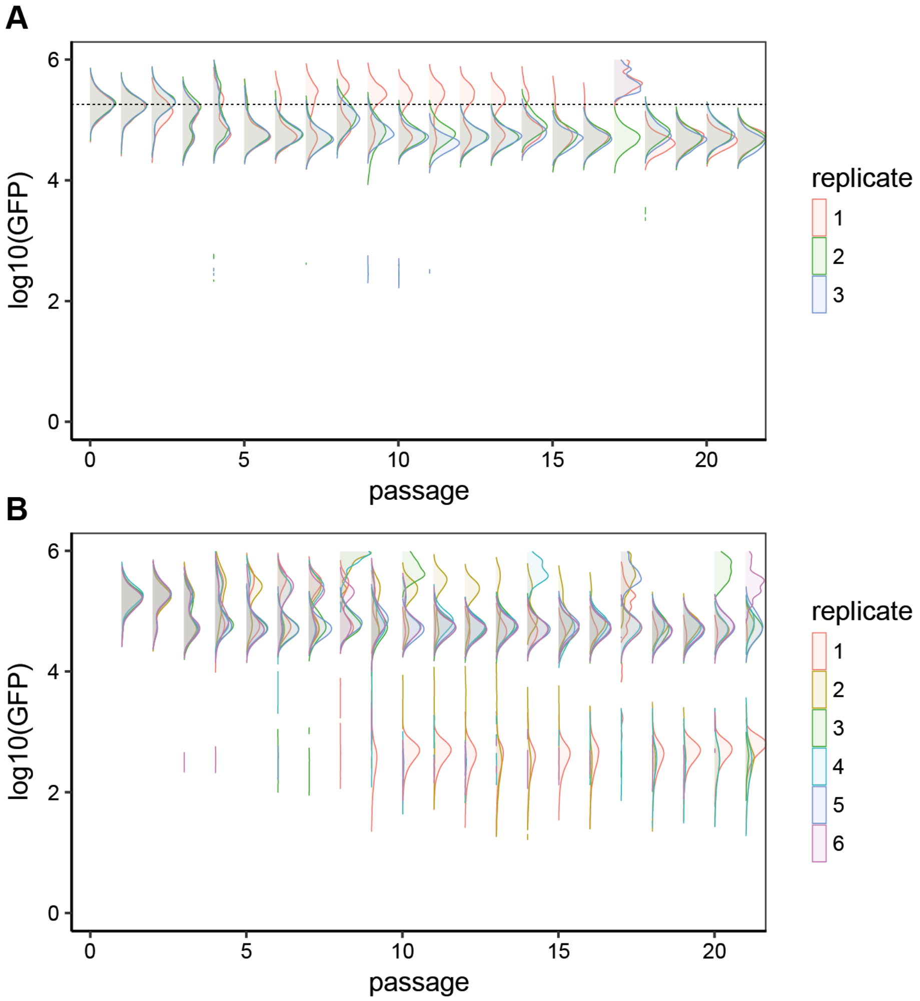

Figure S10: related to Figure 2. Flow cytometry samples of EcN-Lux:ColE1-GFP-HS. A) 3 replicates grown in LB media with erythromycin and kanamycin, ensuring plasmid maintenance. The black dashed line indicates the median fluorescence at passage 0. B) 6 replicates grown in LB media with erythromycin only, allowing for plasmid loss.

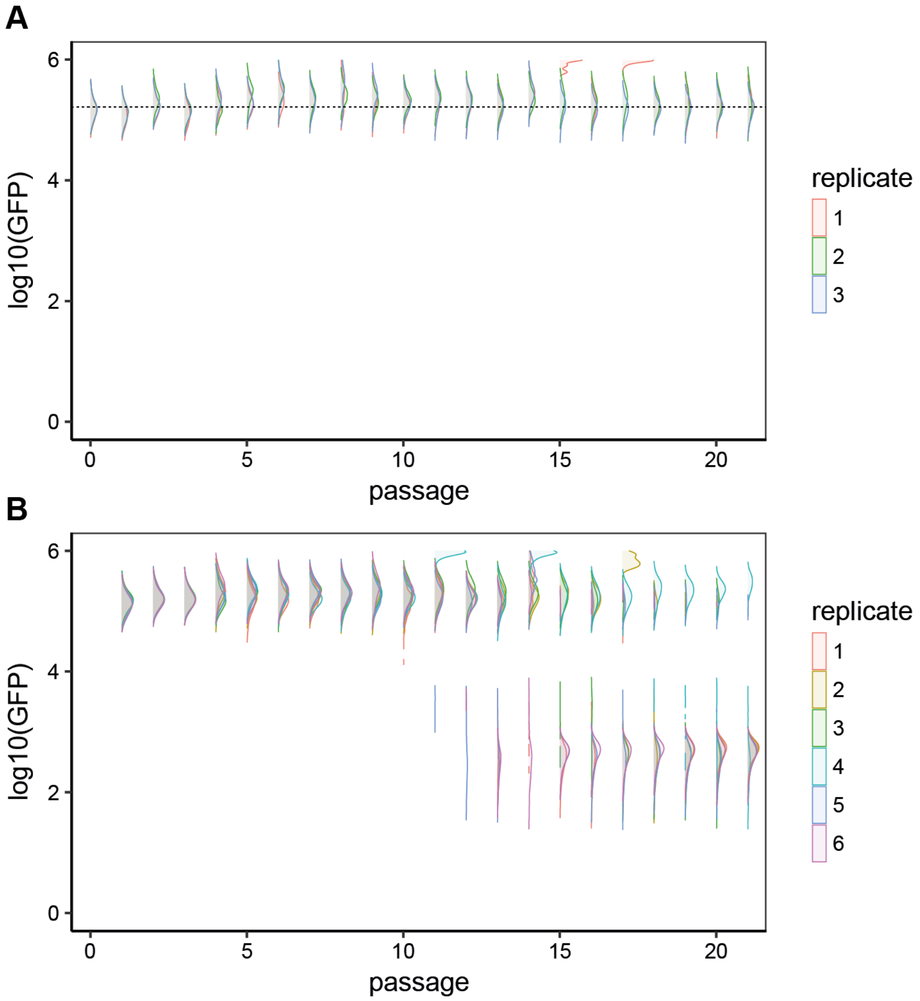

Figure S11: related to Figure 2. Flow cytometry samples of EcN-Lux:pUC-GFP-HS. A) 3 replicates grown in LB media with erythromycin and kanamycin, ensuring plasmid maintenance. The black dashed line indicates the median fluorescence at passage 0. B) 6 replicates grown in LB media with erythromycin only, allowing for plasmid loss.

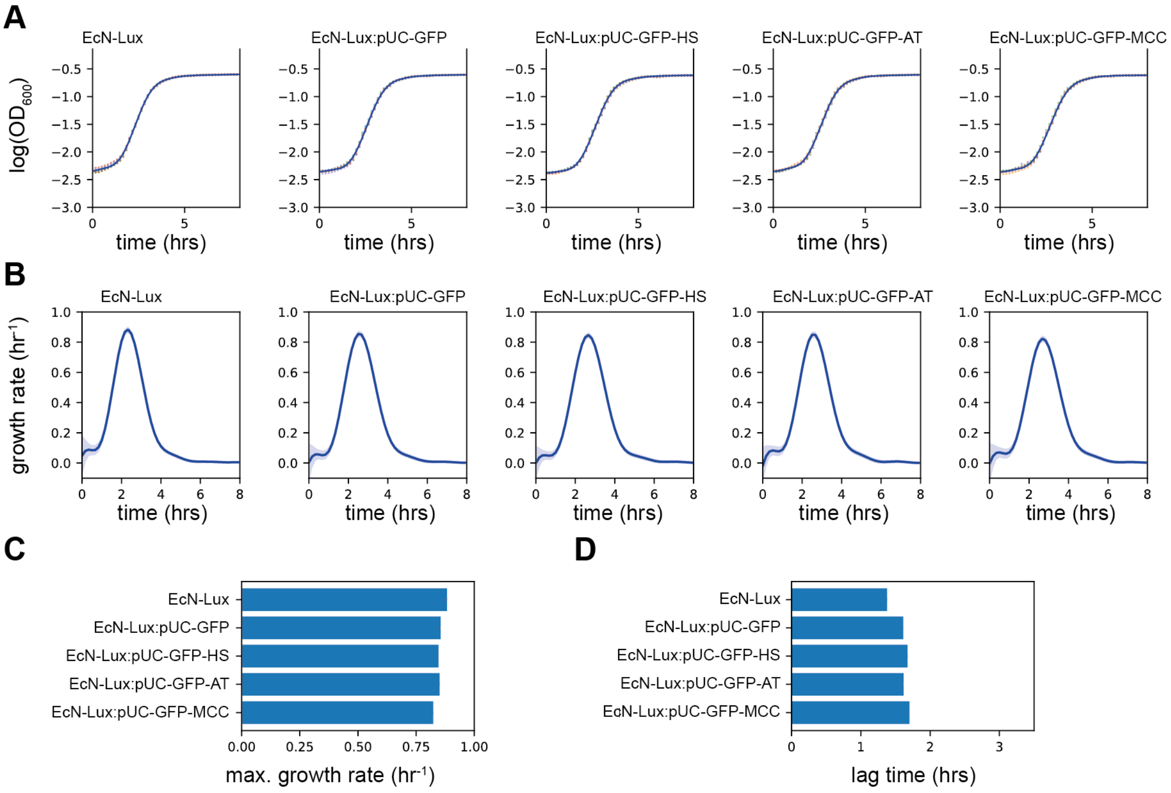

Figure S12: related to Figure 3. Growth curves and model fits for the fluorescent plasmids with different PSK systems in EcN-Lux grown in LB media with erythromycin. (A) Logged optical density measured at 600nm. The points show 6 replicates for each strain with the different colours indicating each replicate. The blue line shows the non-parametric Gaussian process fit with standard error shown by the light blue area around the line. (B) Estimated growth rate as a function of time. (C) Estimated maximal growth rates and (D) estimated lag time. The red lines show the standard deviation of the mean.

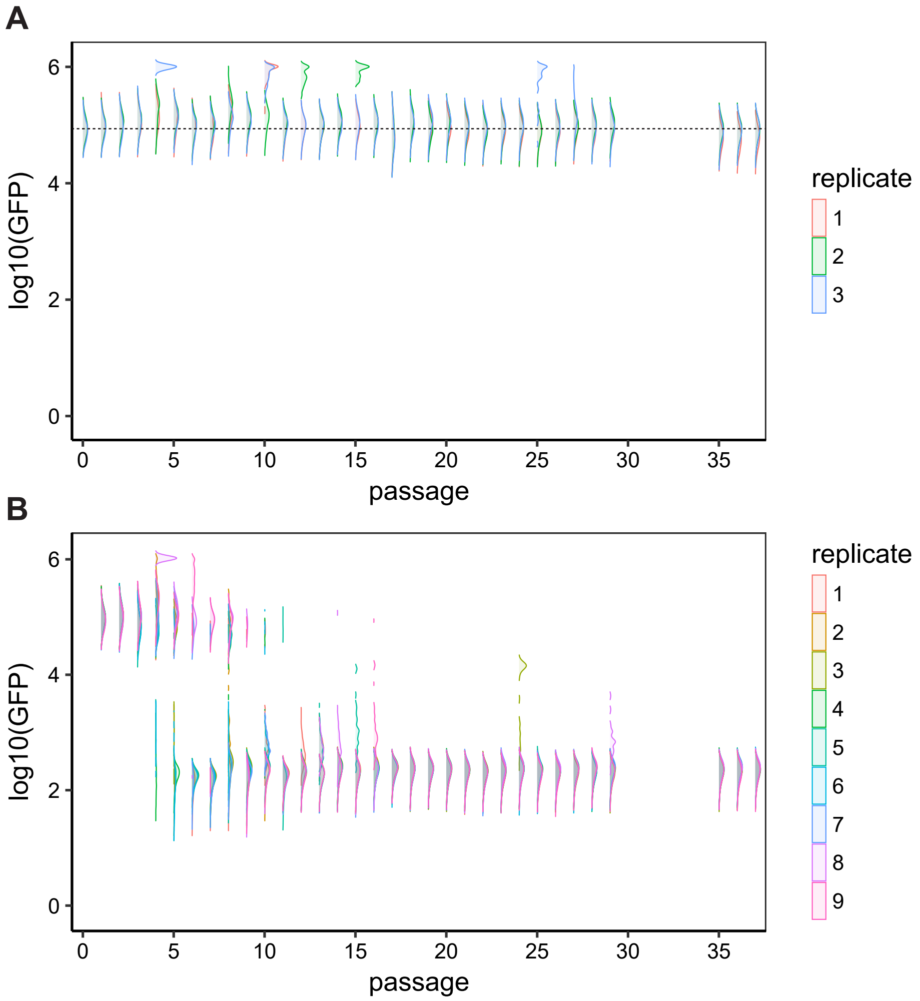

Figure S13: related to Figure 3. Flow cytometry samples of EcN-Lux:pUC-GFP. A) 3 replicates grown in LB media with erythromycin and kanamycin, ensuring plasmid maintenance. The black dashed line indicates the median fluorescence at passage 0. B) 9 replicates grown in LB media with erythromycin only, allowing for plasmid loss.

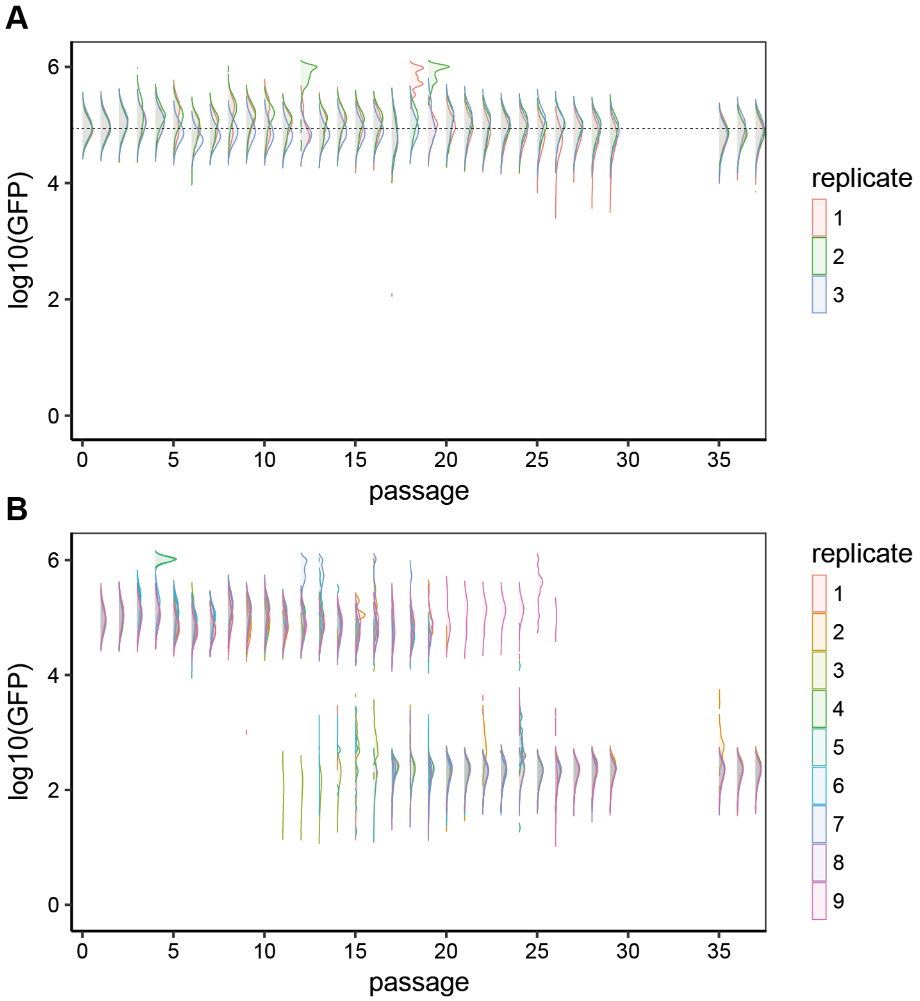

Figure S14: related to Figure 3. Flow cytometry samples of EcN-Lux:pUC-GFP-HS. A) 3 replicates grown in LB media with erythromycin and kanamycin, ensuring plasmid maintenance. The black dashed line indicates the median fluorescence at passage 0. B) 9 replicates grown in LB media with erythromycin only, allowing for plasmid loss.

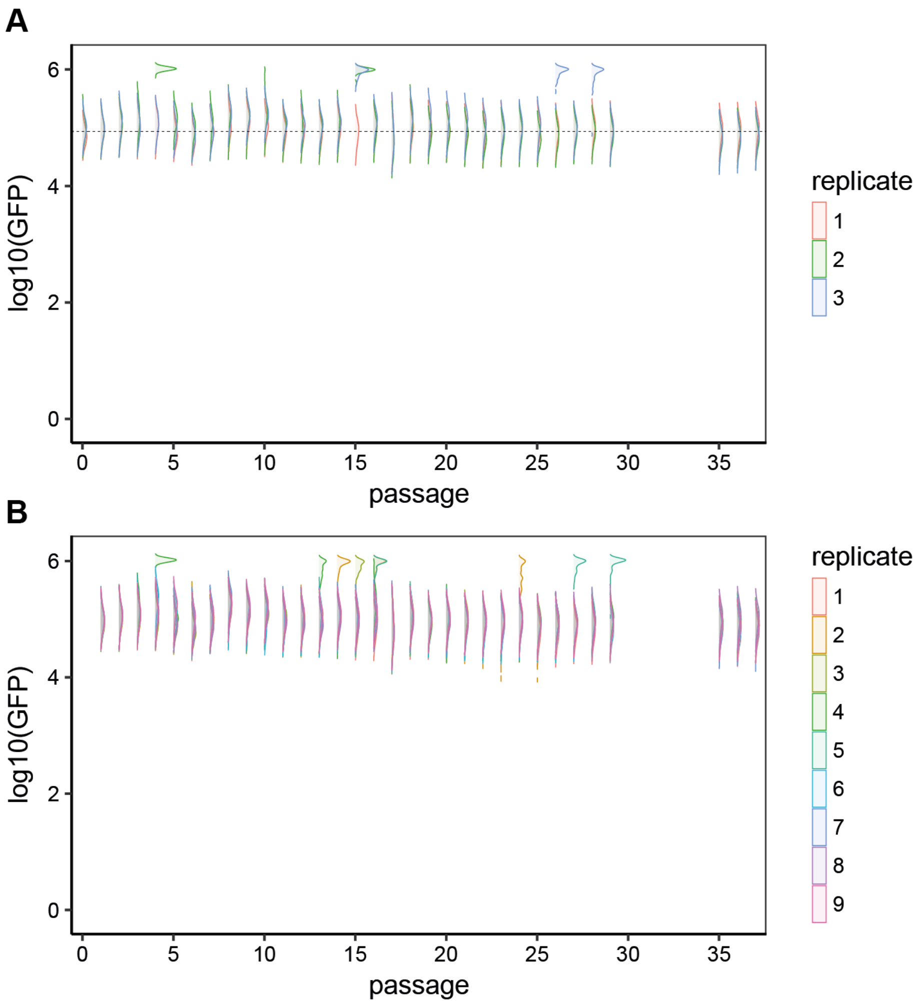

Figure S15: related to Figure 3. Flow cytometry samples of EcN-Lux:pUC-GFP-AT. A) 3 replicates grown in LB media with erythromycin and kanamycin, ensuring plasmid maintenance. The black dashed line indicates the median fluorescence at passage 0. B) 9 replicates grown in LB media with erythromycin only, allowing for plasmid loss.

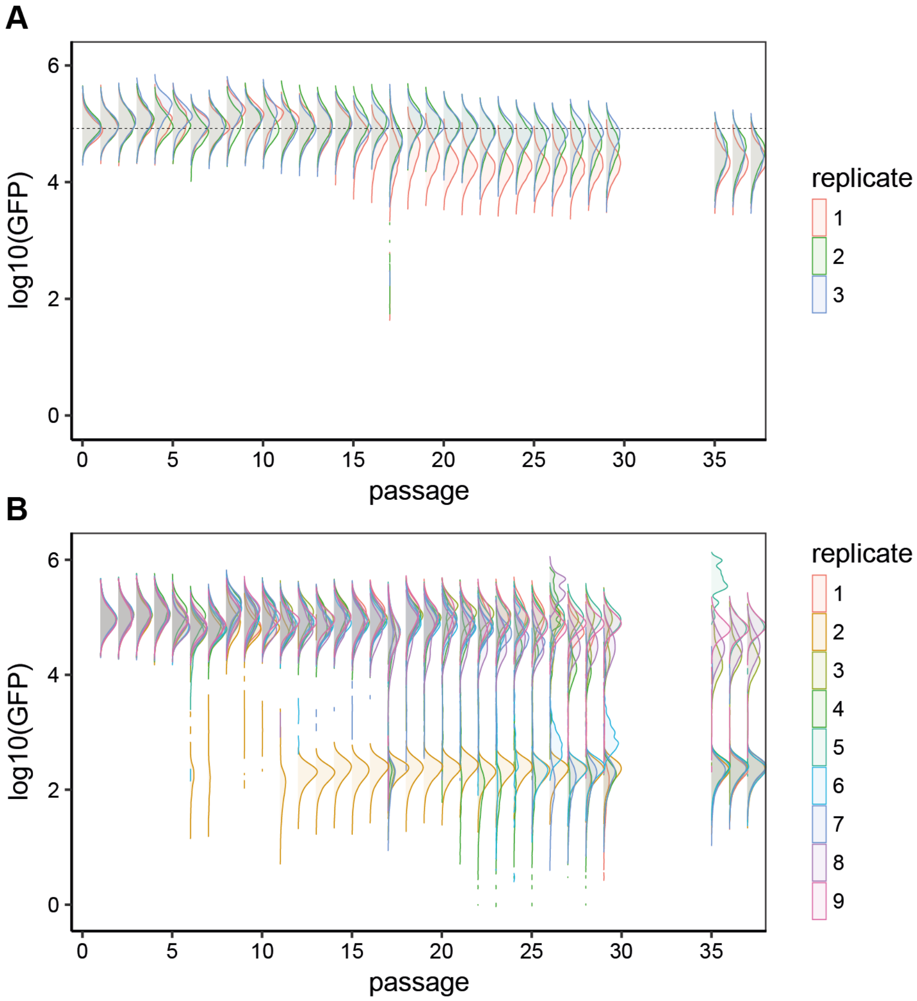

Figure S16: related to Figure 3. Flow cytometry samples of EcN-Lux:pUC-GFP-MCC. A) 3 replicates grown in LB media with erythromycin and kanamycin, ensuring plasmid maintenance. The black dashed line indicates the median fluorescence at passage 0. B) 9 replicates grown in LB media with erythromycin only, allowing for plasmid loss.

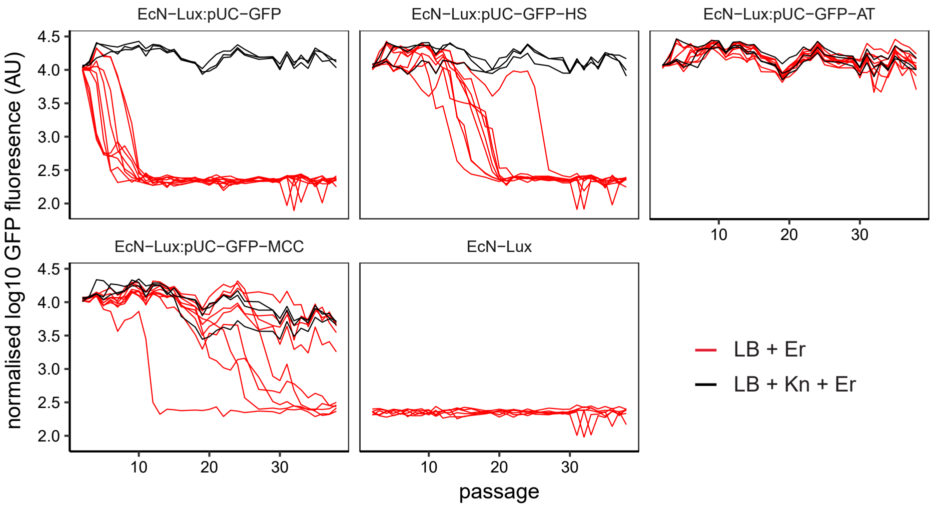

Figure S17: related to Figure 3. GFP fluorescence measurements throughout fluorescent plasmid loss experiment. The black lines (3 replicates) show the trajectories of strains passaged in LB media with erythromycin and kanamycin to ensure plasmid maintenance. The red lines (9 replicates for plasmid-bearing strains and 6 replicates for EcN-Lux control) show the trajectories in LB media with erythromycin but without kanamycin, allowing for plasmid loss. The data is normalised by negating the mean fluorescence of the EcN-Lux in LB+Er (plasmid-free control) at each passage and scaling so that the lowest recorded measurement is equal to 0.

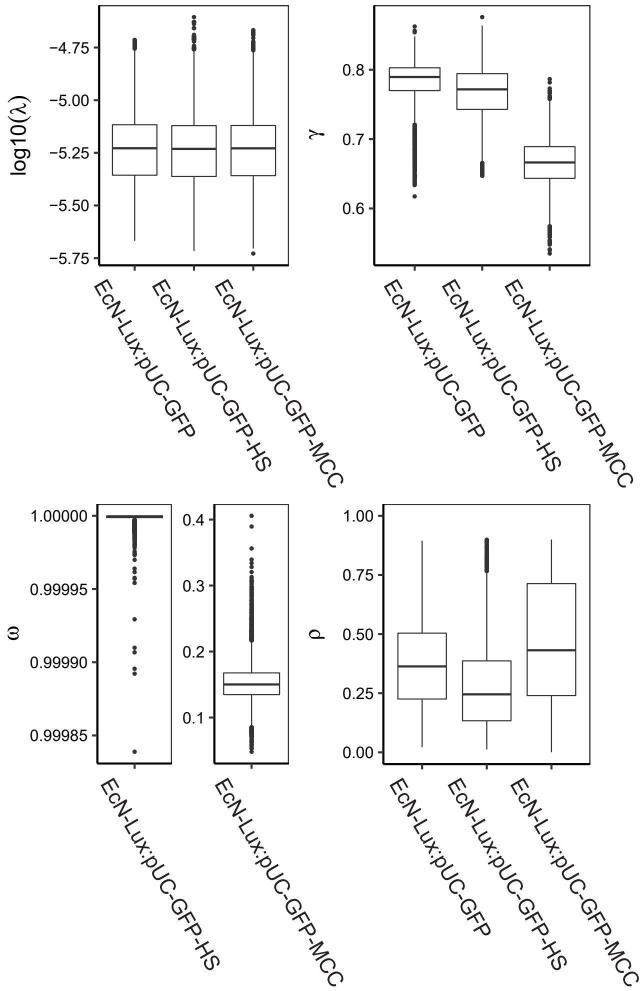

Figure S18: related to Figure 3. Posterior distributions of the model parameters produced from fitting to the plasmid loss curves. EcN-Lux:pUC-GFP has no posterior distribution for ω as it does not carry a PSK system.

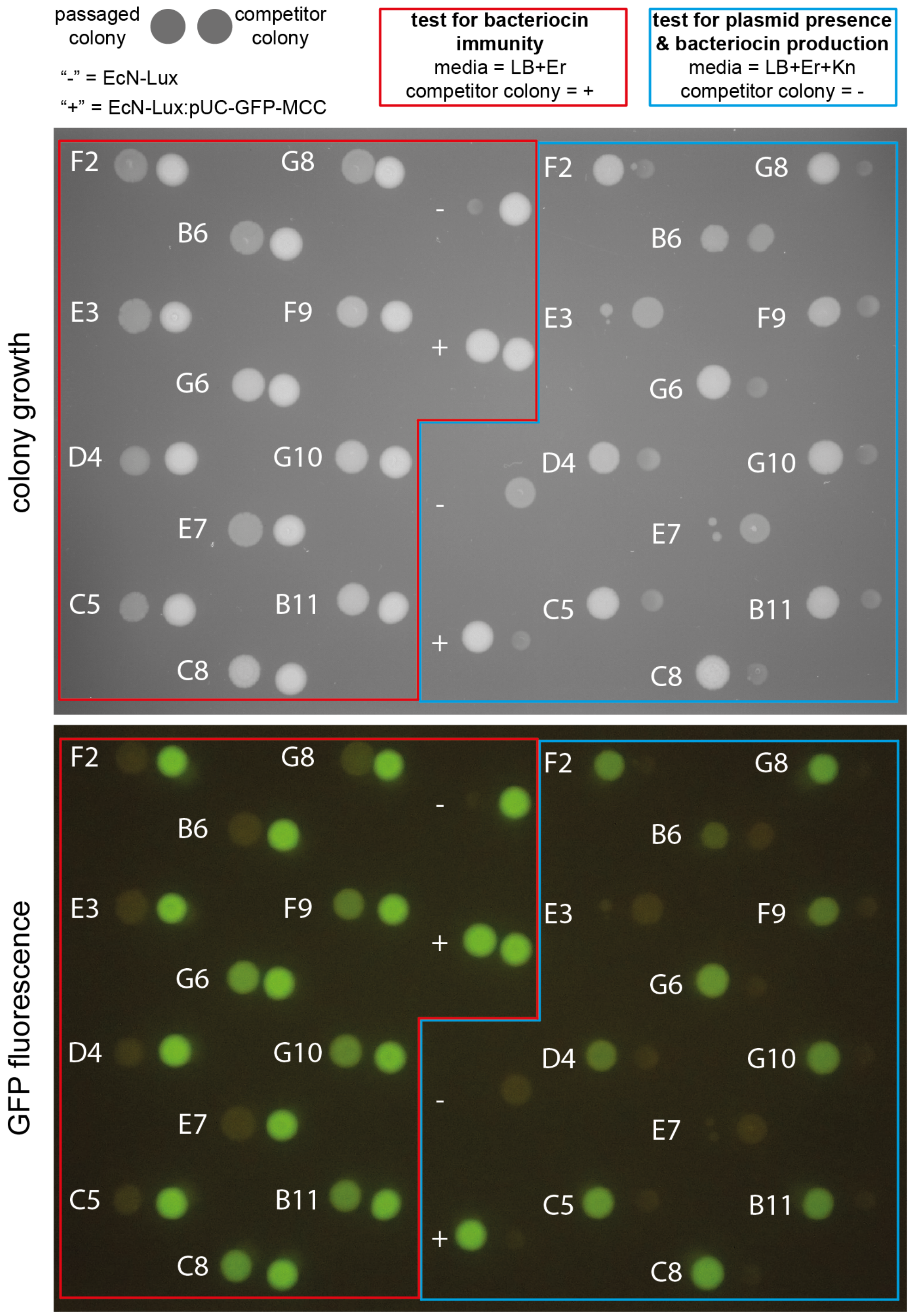

Figure S19: related to Figure 3. Test of bacteriocin production and immunity in EcN-Lux:pUC-GFP-MCC cultures after 30 days of passaged growth. Cultures from the 30th passage of the plasmid loss experiment were grown in LB+Er and LB+Er+Kn and then spotted onto LB+Er agar. The colonies grown in LB+Er were spotted next to a bacteriocin producing colony of EcN-Lux:pUC-GFP-MCC. All of the colonies grow, indicating immunity to the bacteriocin. The negative control, EcN-Lux, shows severely impaired growth. The cultures grown in LB+Er+Kn were spotted next to a bacteriocin sensitive colony, EcN-Lux. Two of the colonies (E3 and E7) show very minimal growth, indicating very limited numbers of plasmid-bearing cells remaining in the source culture. The other colonies show normal growth and increased fluorescence, indicating that the plasmid-bearing cells are still GFP expressing and are being selected for by the kanamycin. All but three of the bacteriocin sensitive colonies show impaired growth. This demonstrates that the plasmid-bearing cells in the source cultures are still producing bacteriocin.

Table S4: related to Figure 3. Summary of results from Figure S19.

Strain	Media	Colony Growth	Competitor Inhibition	Fluorescence
x	LB + Er	Immune to bacteriocin	-	Likely plasmid bearing majority
	LB + Er + Kn	Likely plasmid bearing	Producing bacteriocin	No functional mutation
EcN-Lux:pUC-GFP-MCC passaged in LB + Er	LB + Er	9/9	0/9	3/9
	LB + Er + Kn	7/9	6/9	7/9
EcN-Lux:pUC-GFP-MCC passaged in LB + Er + Kn	LB + Er	3/3	0/3	3/3
	LB + Er + Kn	3/3	3/3	3/3
EcN-Lux -ve control	LB + Er	0/1	0/1	0/1
	LB + Er + Kn	0/1	0/1	0/1
EcN-Lux:pUC-GFP-MCC +ve control	LB + Er	1/1	0/1	1/1
	LB + Er + Kn	1/1	1/1	1/1

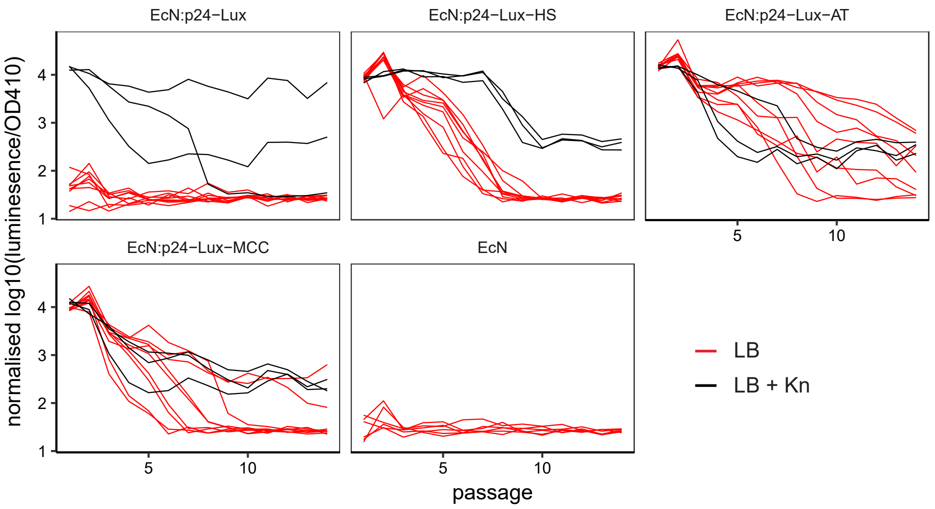

Figure S20: related to Figure 5. Luminescence measurements throughout luminescent plasmid loss experiment. The black lines (3 replicates) show the trajectories of strains passaged in LB media with kanamycin to ensure plasmid maintenance. The red lines (9 replicates for plasmid-bearing strains and 6 replicates for EcN control) show the trajectories in LB media but without kanamycin, allowing for plasmid loss. The data is normalised by negating the mean luminescence of the EcN in LB (plasmid-free control) at each passage and scaling so that the lowest recorded measurement is equal to 0.

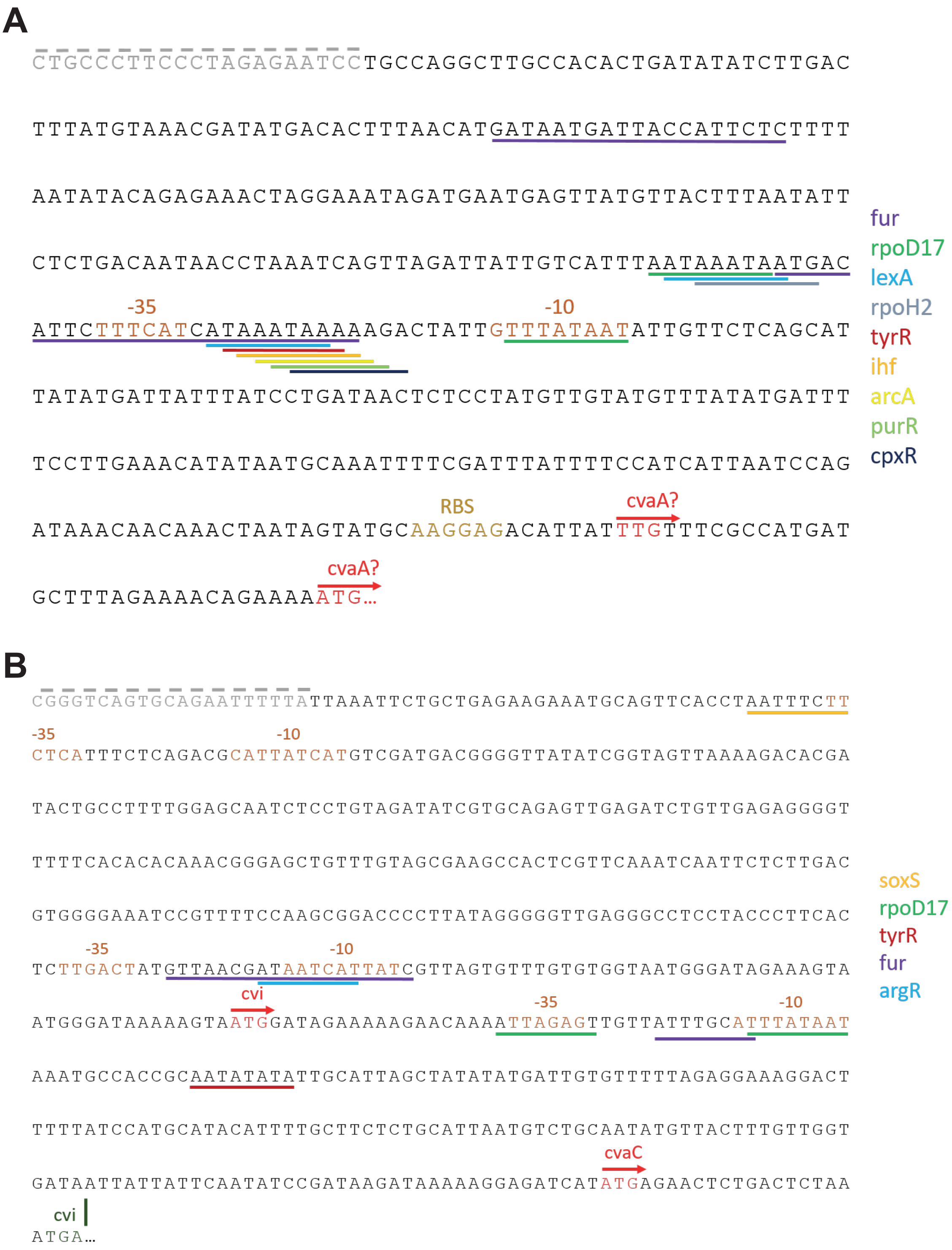

Figure S21: related to Figures 3-5. Upstream regions of microcin-V with -35, -10 and transcription factor recognition sites annotated using BPROM (http://www.softberry.com) A) The region upstream of the cvaA and cvaB genes. Additional annotations of *fur* recognition sites, putative RBS and two potential start codons from Boyer & Tai 1998. B) The region upstream of the cvi and cvaC genes with additional *fur* from Boyer & Tai 1998. Note that there is a putative promoter region after the cvi start codon potentially transcribing the cvaC gene.

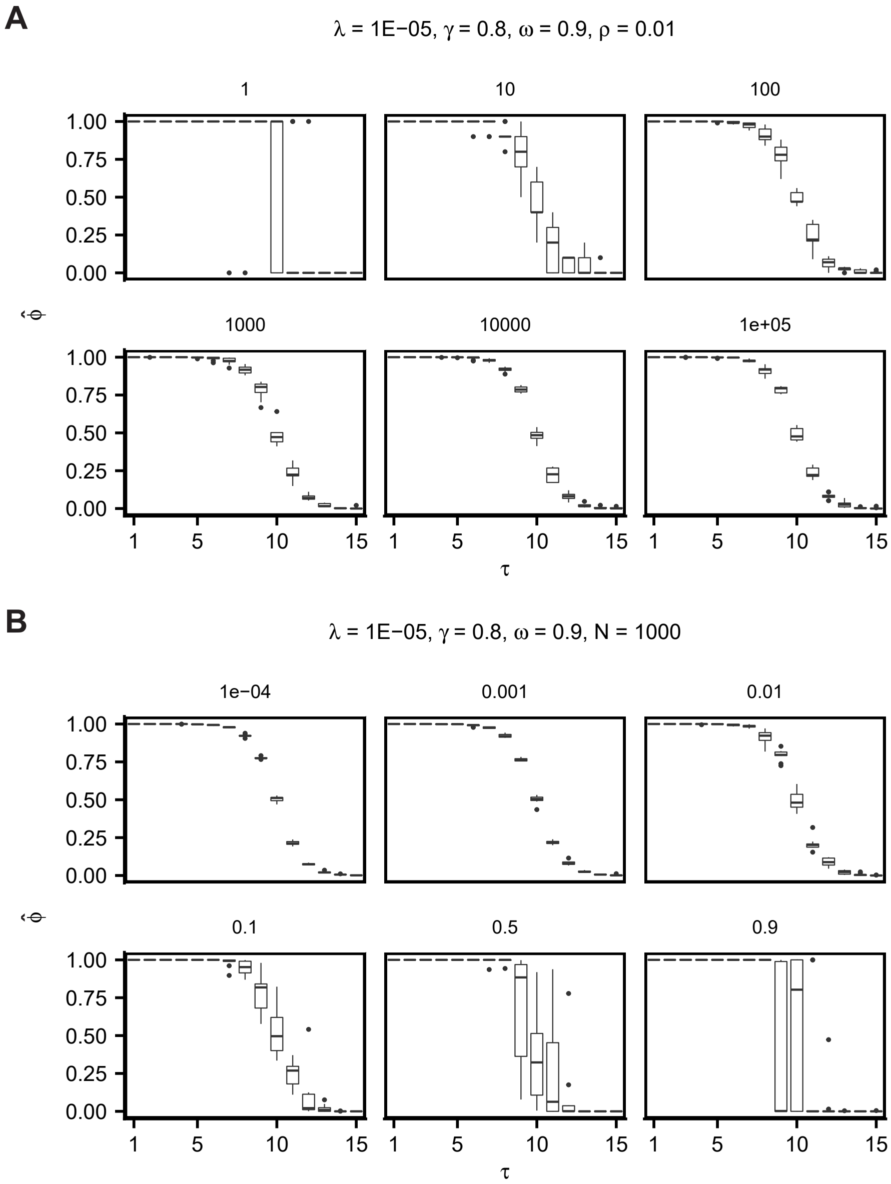

Figure S22: related to Figures 3 and 4. Effects of noise parameters on plasmid-loss curve simulated with the TA model. Nine replicates for each plasmid-loss curve. Varying a) sample size, N, and b) the uncertainty parameter, ρ.

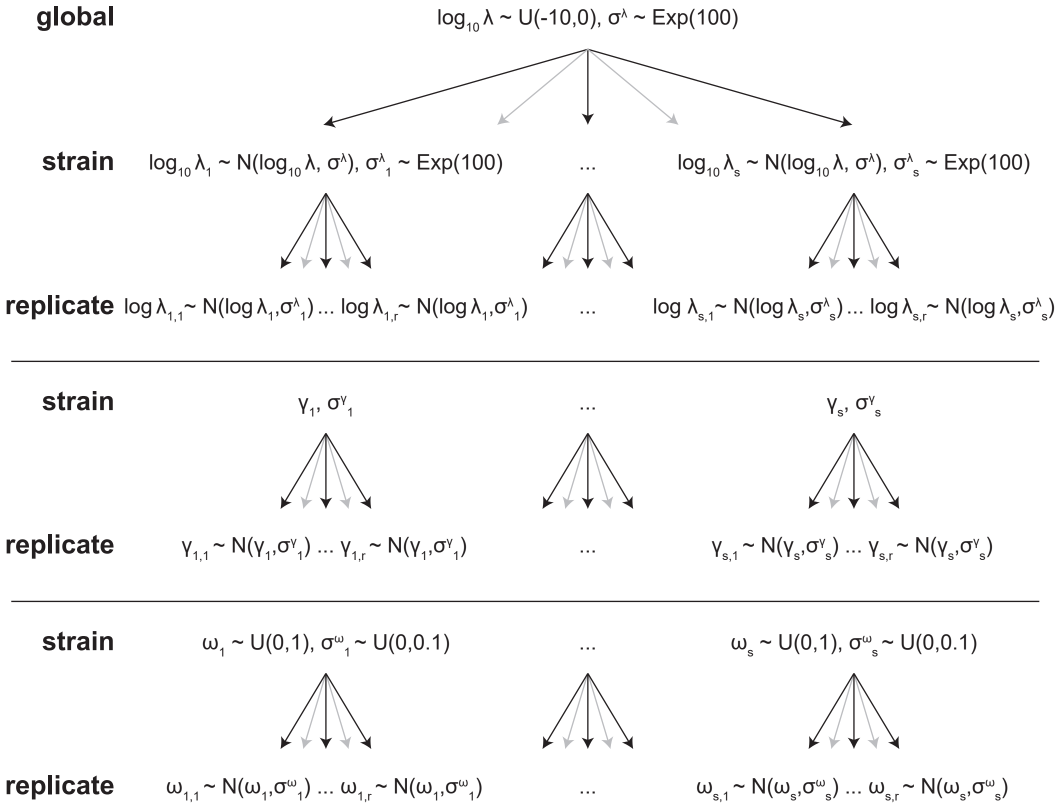

Figure S23: related to Figures 3 and 4. Hierarchical model for fitting parameters of the plasmid loss models to plasmid loss data.

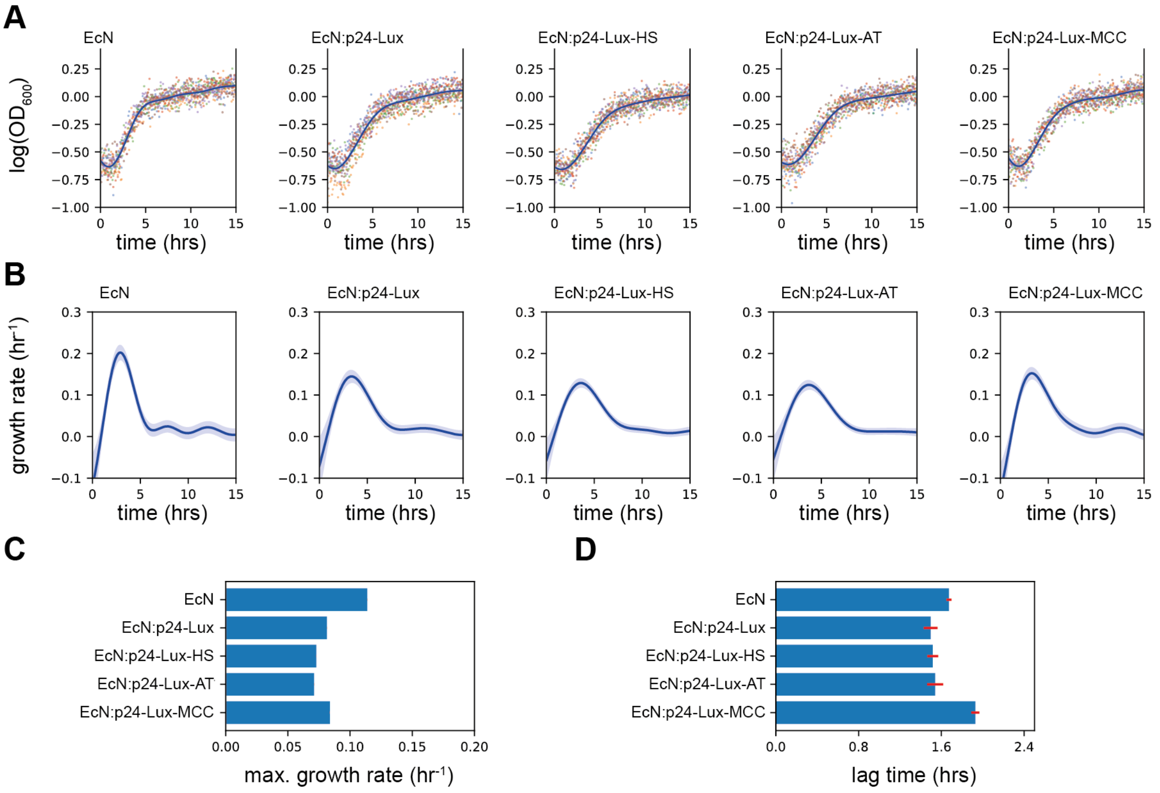

Figure S24: related to Figure 5. Growth curves and model fits for the luminescent plasmids in EcN grown in LB media. For the plasmid bearing strains the media also contained kanamycin to ensure plasmid maintenance. (A) Logged optical density measured at 600nm. The points show 6 replicates for each strain with the different colours indicating each replicate. The blue line shows the non-parametric Gaussian process fit with standard error shown by the light blue area around the line. (B) Estimated growth rate as a function of time. (C) Estimated maximal growth rates and (D) estimated lag time. The red lines show the standard deviation of the mean.

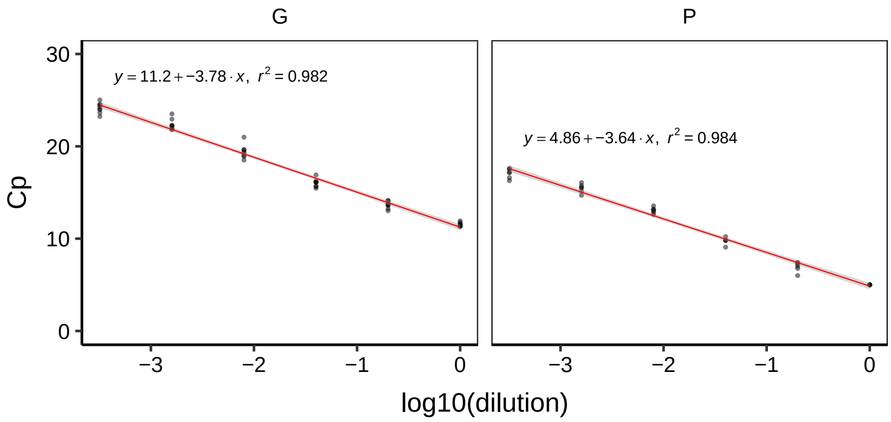

Figure S25: related to Figures 2 and 3. Calibration curves for genomic and plasmid DNA amplification with linear fit (red line).

References

Biernacki, Celeux & Govaert (2000), 'Assessing a mixture model for clustering with the integrated completed likelihood', *IEEE Transactions on Pattern Analysis and Machine Intelligence* **22**(7), 719725.

Carpenter, B., Gelman, A., Hoffman, M., Lee, D., Goodrich, B., Betancourt, M., Brubaker, M. A., Guo, J., Li, P. & Riddell, A. (2016), 'Stan: A probabilistic programming language', *Journal of Statistical Software* **20**, 1–37.

Danino, T., Prindle, A., Kwong, G. a., Skalak, M., Li, H., Allen, K., Hasty, J. & Bhatia, S. N. (2015), 'Programmable probiotics for detection of cancer in urine', *Science Translational Medicine* **7**(289), 289ra84–289ra84.

Gerdes, K. (1988), 'The parB (hok/sok) Locus of Plasmid R1: A General Purpose Plasmid Stabilization System', *Nature Biotechnology* **6**(12), 1402–1405.

Gibson, D. G., Young, L., Chuang, R.-Y., Venter, J. C., Hutchison, C. A. & Smith, H. O. (2009), 'Enzymatic assembly of dna molecules up to several hundred kilobases', *Nature methods* **6**(5), 343–345.

Gilson, Mahanty & Kolter (1987), 'Four plasmid genes are required for colicin v synthesis, export, and immunity.', *Journal of bacteriology* **169**(6), 246670.

Grady, R. & Hayes, F. (2003), 'AxeTxe, a broad-spectrum proteic toxinantitoxin system specified by a multidrug-resistant, clinical isolate of Enterococcus faecium', *Molecular Microbiology* **47**(5), 1419–1432.

Hall, B. G., Acar, H., Nandipati, A. & Barlow, M. (2013), 'Growth rates made easy', *Molecular biology and evolution* **31**(1), 232–238.

Martinez-Garcia, E., Aparicio, T., Goñi-Moreno, A., Fraile, S. & de Lorenzo, V. (2014), 'Seva 2.0: an update of the standard european vector architecture for de-/re-construction of bacterial functionalities', *Nucleic acids research* **43**(D1), D1183–D1189.

Pierce, K. E., Rice, J. E., Sanchez, J. A. & Wangh, L. J. (2002), 'Quantilysetm: reliable dna amplification from single cells', *Biotechniques* **32**(5), 1106–1111.

Riedel, C. U., Casey, P. G., Mulcahy, H., O'Gara, F., Gahan, C. G. M. & Hill, C. (2007), 'Construction of p16slux, a Novel Vector for Improved Bioluminescent Labeling of Gram-Negative Bacteria', *Applied and Environmental Microbiology* **73**(21), 7092–7095.

Sharma, A. (2012), 'An ultraviolet-sterilization protocol for microtitre plates', *Journal of Experimental Microbiology and Immunology (JEMI) Vol* **16**, 144–147.

Silva-Rocha, R., Martinez-Garcia, E., Calles, B., Chavarría, M., Arce-Rodríguez, A., de Las Heras, A., Páez-Espino, A. D., Durante-Rodríguez, G., Kim, J., Nikel, P. I. et al. (2012), 'The standard european vector architecture (seva): a coherent platform for the analysis and deployment of complex prokaryotic phenotypes', *Nucleic acids research* **41**(D1), D666–D675.

Swain, P. S., Stevenson, K., Leary, A., Montano-Gutierrez, L. F., Clark, I. B. N., Vogel, J. & Pilizota, T. (2016), 'Inferring time derivatives including cell growth rates using Gaussian processes', *Nature Communications* **7**, ncomms13766.

Wolfram Research, Inc. (n.d.), 'Mathematica 8.0'.
URL: *https://www.wolfram.com*
